# A role for diatom-like silicon transporters in calcifying coccolithophores

**DOI:** 10.1038/ncomms10543

**Published:** 2016-02-04

**Authors:** Grażyna M. Durak, Alison R. Taylor, Charlotte E. Walker, Ian Probert, Colomban de Vargas, Stephane Audic, Declan Schroeder, Colin Brownlee, Glen L. Wheeler

**Affiliations:** 1 Marine Biological Association, The Laboratory, Citadel Hill, Plymouth, Devon PL1 2PB, UK; 2 Department of Biology and Marine Biology, University of North Carolina Wilmington, 601 South College Road, Wilmington, North Carolina, 28403-5915, USA; 3 Station Biologique de Roscoff, Place Georges Teissier, 29680 Roscoff, France; 4 School of Ocean and Earth Sciences, University of Southampton, National Oceanography Centre, Southampton SO14 3ZH, UK

## Abstract

Biomineralization by marine phytoplankton, such as the silicifying diatoms and calcifying coccolithophores, plays an important role in carbon and nutrient cycling in the oceans. Silicification and calcification are distinct cellular processes with no known common mechanisms. It is thought that coccolithophores are able to outcompete diatoms in Si-depleted waters, which can contribute to the formation of coccolithophore blooms. Here we show that an expanded family of diatom-like silicon transporters (SITs) are present in both silicifying and calcifying haptophyte phytoplankton, including some globally important coccolithophores. Si is required for calcification in these coccolithophores, indicating that Si uptake contributes to the very different forms of biomineralization in diatoms and coccolithophores. Significantly, SITs and the requirement for Si are absent from highly abundant bloom-forming coccolithophores, such as *Emiliania huxleyi*. These very different requirements for Si in coccolithophores are likely to have major influence on their competitive interactions with diatoms and other siliceous phytoplankton.

## Abstract (web summary)

Silicification by diatoms and calcification by coccolithophores are distinct biomineralisation processes that influence global carbon cycling and the abundance of marine plankton. Here, Durak *et al*. show that diatom-like silicon transporters are present in coccolithophores, and that silicon is required for formation of their calcium carbonate coccoliths.

## Graphical abstract

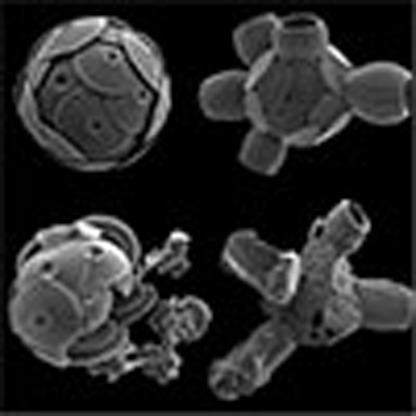

The biomineralized phytoplankton are major contributors to marine primary productivity and play a major role in carbon export to the deep oceans by promoting the sinking of organic material from the photic zone^1^,^2^. The two primary forms of biomineralization found in marine plankton are the precipitation of silica (by diatoms, chrysophytes, synurophytes, dictyochophytes, choanoflagellates and radiolarians) and calcium carbonate (by coccolithophores, foraminifera, ciliates and dinoflagellates)^3^. These processes require very different chemistries and exhibit no known shared mechanisms. Both silicification and calcification appear to have evolved independently on multiple occasions. However, since in many cases the underlying cellular mechanisms have not been elucidated, the evolutionary processes remain unclear. Improved knowledge of the cellular mechanisms of biomineralization will allow us to understand the impact of past climatic events on the major phytoplankton lineages and better predict their response to future environmental change.

The haptophyte algae are of particular interest in the evolution of biomineralization as they include closely related silicified and calcified representatives. The coccolithophores (Calcihaptophycidae)^4^ produce an extracellular covering of ornate calcium carbonate plates (coccoliths) and are major contributors to biogenic calcification in the ocean^3^. The most abundant coccolithophore species in modern oceans are *Emiliania huxleyi* and *Gephyrocapsa oceanica*, which belong to the Noelarhabdaceae. These species have a small cell size and are able to form extensive blooms. Larger coccolithophores species such as *Coccolithus braarudii* and *Calcidiscus leptoporus* are less numerous, but as they are heavily calcified they are important contributors to global calcification^5^. Much of our understanding of coccolithophore biology comes from the study of *E. huxleyi*, but emerging evidence suggests that there is considerable physiological diversity among coccolithophores^6^.

Though the biomineralized haptophytes are predominately calcified, a representative was recently described, *Prymnesium neolepis* (formerly *Hyalolithus neolepis*), which is covered with silica scales and resembles a ‘silicified coccolithophore'^7^,^8^,^9^. The silica scales are produced intracellularly and then deposited outside the plasma membrane, in a manner analogous to coccolith secretion^8^,^10^. The Prymnesiales are estimated to have diverged from the coccolithophores around 280 Myr ago^11^ and *P. neolepis* is the only known extensively silicified haptophyte. Understanding whether common cellular mechanisms contribute to silica scale production in *P. neolepis* and coccolith formation in the coccolithophores may help us to understand how these different forms of biomineralization have evolved in the haptophytes and also in other phytoplankton lineages.

Silicification by marine phytoplankton has both contributed to and been influenced by the marked changes in the biogeochemistry of Si in the surface ocean. The diatoms, representing the dominant silicifying phytoplankton in current oceans, appeared only relatively recently in the fossil record (120 Myr ago) and their expansion in the Cenozoic resulted in the extensive depletion of silicate from the surface ocean, leading to the decline of heavily silicified sponges and decreased silicification in radiolarians^12^,^13^,^14^,^15^. Si has therefore become a limiting nutrient for modern silicifying phytoplankton and is an important factor in competitive interactions with non-silicifying taxa. As the regeneration of available Si from silica dissolution is slow, diatom blooms can deplete Si in the surface ocean sufficiently to prevent further growth. If other nutrients such as nitrate or phosphate are still available, then Si limitation can contribute to seasonal succession, where an initial diatom spring bloom is followed by subsequent blooms of non-siliceous phytoplankton. There is evidence that the low availability of Si is an important contributory factor in the formation of some coccolithophore blooms. Major *E. huxleyi* blooms in areas such as the North Atlantic, the Black Sea and off the Patagonian shelf have been associated with low silicate availability^16^,^17^,^18^. These observations support the view that the ecological niche of coccolithophores is partly defined by conditions that reduce competition with the fast-growing resource-efficient diatoms, such as in areas of low silicate where other nutrients (for example, nitrate and phosphate) remain available^19^.

To further understand the evolution of biomineralization in haptophytes, we characterized the cellular mechanisms underlying silica scale formation in *P. neolepis*. We examine commonalities with other silicified organisms and determine whether any common cellular mechanisms contribute to biomineralization in silicified and calcified haptophytes. Surprisingly, given that it is generally assumed that coccolithophores lack a requirement for Si, we identify that diatom-like Si transporters are present in haptophytes, not only in the silicified *P. neolepis* but also in some important calcifying coccolithophore species. We demonstrate that Si plays an important role in formation of calcite coccoliths in these coccolithophores, but that the requirement for Si is significantly absent from the most abundant species in present day oceans, *E. huxleyi*. The findings have important implications for the evolution of the biomineralized phytoplankton and their distribution in both past and modern oceans.

## Results

### Mechanisms of biomineralization in a silicifying haptophyte

The known mechanisms of biosilicification in eukaryotes involve a number of common elements; a mechanism for Si uptake, an acidic silica deposition vesicle and an organic matrix for catalysing and organizing silica precipitation^20^. However, there is little evidence for shared mechanisms at the molecular level, suggesting that silicification has evolved independently in many lineages. We therefore examined the mechanisms of silicification in *P. neolepis*, using both molecular and physiological approaches. At low Si concentrations, Si uptake in diatoms is performed by a family of Na^+^-coupled high-affinity Si transporters (SITs), although diatoms may also acquire Si by diffusive entry at higher Si concentrations^21^,^22^. Silicified sponges and land plants do not contain SITs, but use alternative mechanisms for Si transport^23^,^24^. A search for putative Si transporters in the transcriptome of *P. neolepis* strain PZ241 (Supplementary Fig. 1) identified a single gene bearing similarity to the SITs (*PnSIT1*). PnSIT1 exhibits 24.9–29.3% identity and 39.8–47.0% similarity to diatom SITs at the amino-acid level (sequences used for comparison were *Cylindrotheca. fusiformis* AAC49653.1*, Thalassiosira. pseudonana* ABB81826.1 and *Phaeodactylum tricornutum* ACJ65494.1). SITs have only previously been identified in siliceous stramenopiles (diatoms and chrysophytes) and choanoflagellates^25^,^26^,^27^. Many features of PnSIT1 are conserved with these SITs, including the 10 predicted transmembrane regions and the pair of motifs (EGxQ and GRQ) between TM2-3 and TM7-8 (ref. 27; Supplementary Fig. 2). We also identified a homologue of the Si efflux protein, Lsi2 in *P. neolepis* (Supplementary Table 1). Lsi2 is related to the bacterial arsenate transporter ArsB and mediates Si efflux in plant cells^28^. Lsi2 is also present in diatoms and its transcriptional regulation is highly similar to SIT2 in *Thalassiosira pseudonana*, although its cellular role has not yet been characterized^29^. The identification of Lsi2 in *P. neolepis* suggests that it may play a conserved role in siliceous phytoplankton.

**Figure 1:**
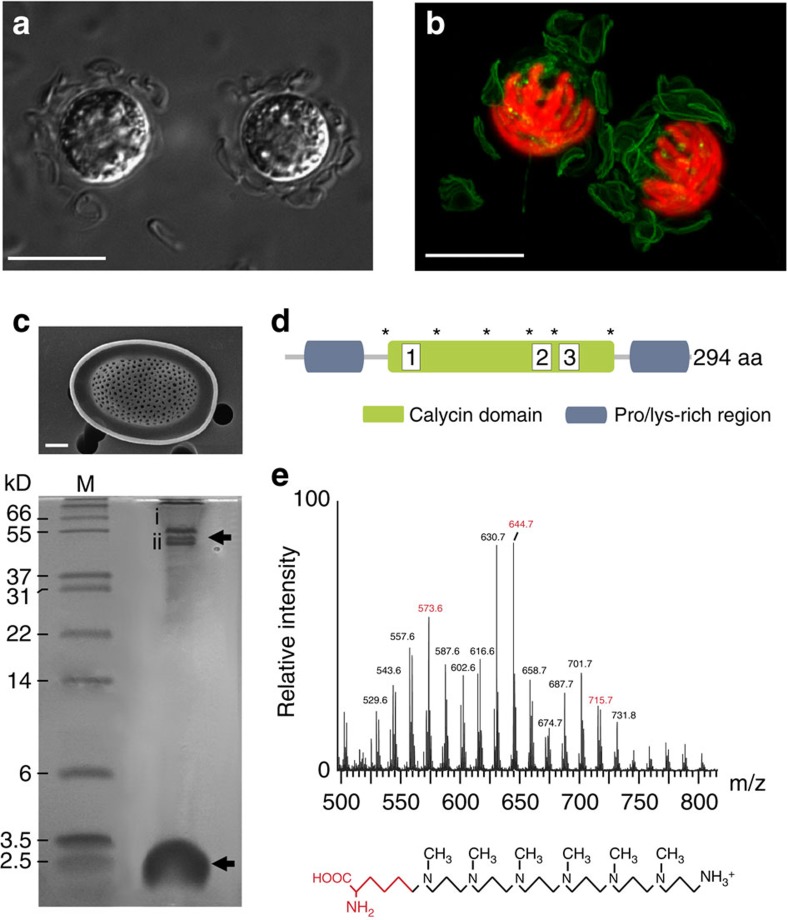
Molecular mechanisms of silica scale production in *P. neolepis*. (**a**) Differential interference contrast (DIC) microscopy image of *P. neolepis* cells displaying the loose covering of silica scales. Scale bar, 10 μm. (**b**) Confocal microscopy of a *P. neolepis* cell showing incorporation of the fluorescent dye HCK-123 into newly formed silica scales (green). Chlorophyll autofluorescence is shown in red. The 3D-projection was generated from compiling a Z-stack of 15 images. Scale bar, 10 μm. (**c**) Tricine/SDS–PAGE of organic components released after dissolution of silica scales with NH_4_F. A SEM image of an isolated silica scale is also shown (Scale bar, 1 μm). The higher molecular weight component around 50 kDa is a single protein that runs as two bands (i, ii), whereas the low-molecular-weight components around 2.5 kDa are long-chain polyamines (LCPA). M, molecular-weight markers. (**d**) Domain organization of the lipocalin-like protein (LPCL1) identified from both protein bands in NH_4_F extracted silica scales. The approximate positions of the proline/lysine-rich regions and the calycin domain (IPR012674) are shown. Also shown are the positions of six highly conserved cysteines (asterisk) that may be involved in the formation of disulphide bridges. (**e**) Long-chain polyamines (LCPAs) from *P. neolepis* silica scales. Electrospray ionization mass spectrometry (ESI-MS) of the low-molecular-weight NH_4_F-soluble fraction of silica scales revealed a series of mass peaks separated by 71 Da (highlighted in red), characteristic of *N*-methyl propyleneimine units. The additional mass peaks ±14 Da may indicate different methylation states, as is commonly observed in LCPAs. The proposed structure of the LCPAs in *P. neolepis* is shown with the putative lysine residue is highlighted in red.

We next determined the presence of an acidic silica deposition vesicle in *P. neolepis* using the fluorescent dye HCK-123, which partitions into acidic compartments and labels nascent silica (Fig. 1a,b). We found that newly formed silica scales are secreted at the posterior pole of the cell, indicating that the principal cellular components involved in scale formation (silica precipitation in acidic non-Golgi-derived vesicles) are distinct from those involved in coccolith formation (calcite precipitation in alkaline Golgi-derived vesicles and secretion at the anterior pole of the cell)^10^,^30^.

A search of the *P. neolepis* transcriptome for mechanisms involved in silica precipitation did not reveal homologues of any of the known silica-associated proteins from diatoms (silaffins, pleuralins and frustulins) or sponges (silicateins)^20^,^31^ (Supplementary Table 1). As some of these proteins have a low complexity amino-acid composition and may not be identified by sequence similarity searches, we directly analysed the organic components released by NH_4_F dissolution of the silica scales. We identified two major organic components using Tricine/SDS–PAGE; a lipocalin-like protein and long-chain polyamines (LCPAs; Fig. 1c). The lipocalin-like protein contains two proline-/lysine-rich regions surrounding a lipocalin domain and represents a novel silica-associated protein (LPCL1, Fig. 1d, Supplementary Fig. 3). The LCPAs from *P. neolepis* are composed of *N*-methylated oligopropyleneimine repeats, similar to the silica-associated LCPAs previously characterized from diatoms and sponges^32^,^33^, but differ from these LCPAs as the repeat units are linked to a lysine residue rather than putrescine, ornithine or spermidine (diatoms), or butaneamine (sponges) residues (Fig. 1e, Supplementary Fig. 4). Diatoms possess a series of unusual orthologues of the genes involved in polyamine synthesis that are proposed to play a specific role in the formation of LCPAs^34^. Homologues of these modified genes for polyamine synthesis were not found in the *P. neolepis* transcriptome, indicating that these modifications may be specific to diatoms.

### An expanded family of SITs in haptophytes

**Figure 2:**
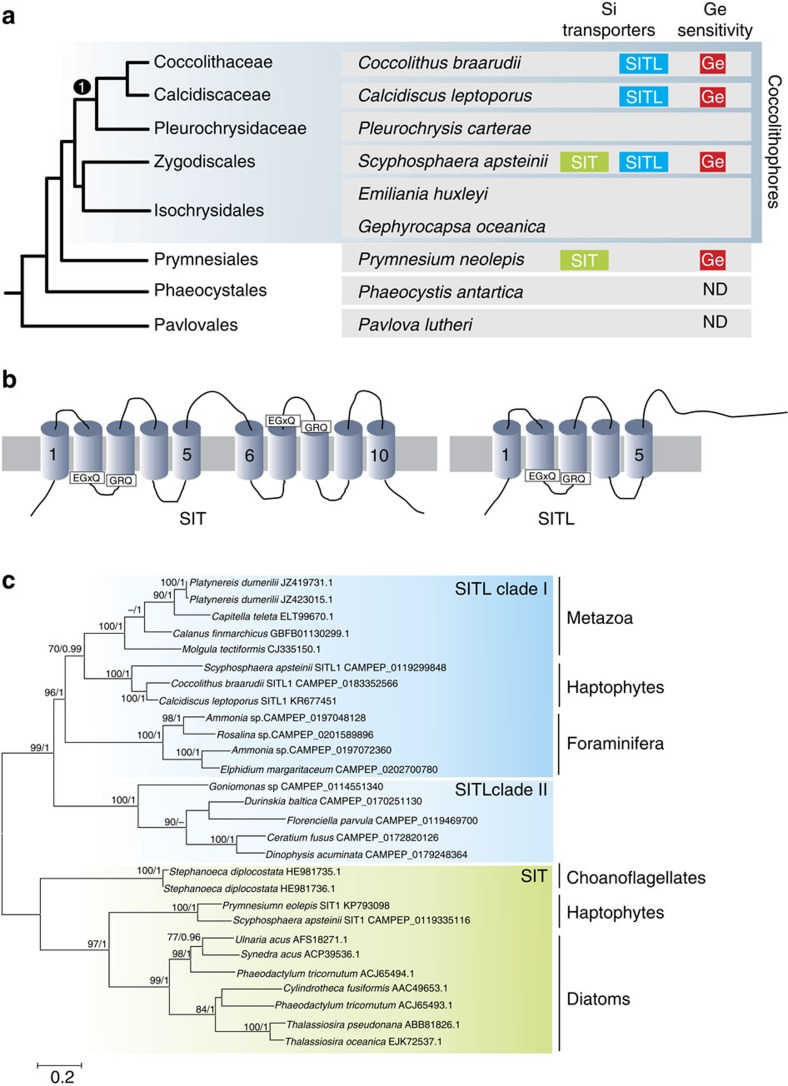
An expanded family of diatom-like Si-transporters (SITs) in haptophytes. (**a**) Phylogenetic relationships between haptophytes. The schematic tree shows the currently accepted phylogenetic relationships of the major haptophyte lineages based on multigene phylogenies^11^. Representative species of each group are indicated, along with the presence of SITs or SITLs in these species. Sensitivity to Ge is shown in red, ND, not determined. Coccolithales. (**b**) A schematic image of the domain architecture of the SITs and the SITLs indicating the approximate position of the transmembrane domains and of the conserved motifs. (**c**) A maximum likelihood phylogenetic tree based on an alignment of selected SITL proteins with SITs (aligned to the N-terminal SIT domain). Final alignment size was 157 amino acids. The SITLs form a well-supported monophyletic clade. Within the SITLs two distinct clades can be observed. SITL clade I contains haptophytes, metazoa and foraminifera, whereas SITL clade II contains dinoflagellates, a cryptophyte and a dictyochophyte. Bootstrap values >70% (100 bootstraps) and Bayesian posterior probabilities >0.95 (10,000,000 generations) are shown above nodes. Scale bar, substitutions per site.

Our analyses indicate that there are some similarities in the biosilicification mechanisms between *P. neolepis* and diatoms, including the silica deposition vesicle and the LCPAs. However, the silica-associated proteins bear no similarity and the only known silicification-related gene products common to both organisms are the Si transporters (SITs and Lsi2). To examine the origins of SITs and Lsi2 in *P. neolepis*, we performed sequence similarity searches of the *Emiliania huxleyi* genome and 24 other haptophyte transcriptomes (including six species of coccolithophore) from the Marine Microbial Eukaryote Transcriptome data set (http://marinemicroeukaryotes.org/)^35^. Homologues of the Si-associated protein LPCL1 from *P. neolepis* were not found in other haptophytes (Supplementary Tables 1 and 2). However, we identified a SIT homologue in the calcifying coccolithophore *Scyphosphaera apsteinii* that was highly similar to PnSIT1 (66% identity, 76.2% similarity at the amino-acid level). In addition, we found that three coccolithophores (*S. apsteinii*, *Coccolithus braarudii* and *Calcidiscus leptoporus*) possess a SIT-like protein that only contains five transmembrane regions (Fig. 2a,b). The Si efflux protein Lsi2 was not found in these coccolithophores, or in any other haptophyte, with the exception of the non-mineralized prymnesiophyte, *Haptolina ericina*.

Comparison of the two haptophyte SITs with 33 other SIT sequences originating from diatoms, chrysophytes and choanoflagellates indicated that all of the highly conserved amino-acid residues identified by Marron *et al*.^26^ were also conserved in haptophytes (Supplementary Fig. 2). The single 5TM domain of the SIT-like (SITL) proteins displays a high sequence similarity to the N- and C-terminal 5TM domains of SITs. SITLs also possess the highly conserved EGxQ and GRQ motifs that are proposed to play a role in binding Si^26^,^27^, as well as many of the other amino-acid residues that were identified as being highly conserved in SITs (Supplementary Fig. 2). The 5TM+5TM inverted repeat topology of the SITs is characteristic of Na^+^-coupled transporters with a LeuT fold and is also found in many other membrane transporters^36^,^37^. The inverted repeat topology in these transporters is thought to have evolved following gene duplication and fusion of a related transporter that initially existed as a homodimer with inverted symmetry^38^. Homodimerization of the SITLs may therefore result in a membrane transporter with similar properties to the SITs and it is likely that SITs evolved from a protein resembling the SITLs. SITLs were not found in any other haptophytes or in diatoms, but were present in a range of other eukaryotes, including foraminifera, dinoflagellates and metazoa (such as the polychaetes, *Capitella teleta* and *Platynereis dumerilii* and the copepod *Calanus finmarchicus*) (Fig. 2c). Many calanoid copepods have silicified teeth^39^ and SITLs may provide a mechanism for Si transport in these ecologically important zooplankton. However, not all of the species that possess SITLs are silicified. The foraminifera and the coccolithophores are the predominant contributors to calcification in our oceans and so the identification of SITLs in these lineages is particularly intriguing.

The SITs from *P. neolepis* and *S. apsteinii* form a strongly supported monophyletic clade, suggesting a common evolutionary origin for the haptophyte SITs (Fig. 2c, Supplementary Fig. 5). To explain the limited distribution of SITs, Marron *et al*.^26^ proposed that horizontal gene transfer (HGT) of SITs may have occurred between stramenopiles and choanoflagellates. However, there is no phylogenetic evidence to support recent HGT of SITs between stramenopiles, choanoflagellates or the haptophytes. The SITL proteins form a monophyletic clade distinct from true SITs, suggesting that they represent a novel but closely related group of transporter proteins (Fig. 2c). When aligned to the SITLs, the individual N- and C-terminal regions of SITs form strongly supported clades, suggesting that the SITs found in stramenopiles, choanoflagellates and haptophytes arose from a single gene duplication event, rather than from a series of more recent duplication events in each lineage (Supplementary Fig. 5). Phylogenetic analyses of Lsi2 provided no indication that haptophytes acquired this gene by recent HGT (Supplementary Fig. 6).

### A novel role for Si in coccolithophore calcification

Calcified coccolithophores emerged in the early Mesozoic (c. 220 Myr ago)^11^, when Si concentrations in the surface oceans were considerably higher than in present day. The distribution of the SITs and SITLs in haptophytes suggests that these transporters were present in ancestral haptophytes, including the last common ancestor of the coccolithophores. Although Si has not been generally identified as a component of calcite coccoliths, a recent study showed that Si is a minor component of the two forms of heterococcolith (muroliths and lopadoliths) found in *S. apsteinii*^40^. In many calcifying systems, calcite precipitation occurs by the crystallization of amorphous calcium carbonate (ACC). Recent evidence indicates that silica can modulate the crystallization of calcium carbonate *in vitro* by acting to modulate the metastability of ACC and facilitate ordered calcite crystal formation^41^,^42^,^43^,^44^. We therefore hypothesized that Si uptake via SITs or SITLs may contribute to calcification in coccolithophores.

**Figure 3:**
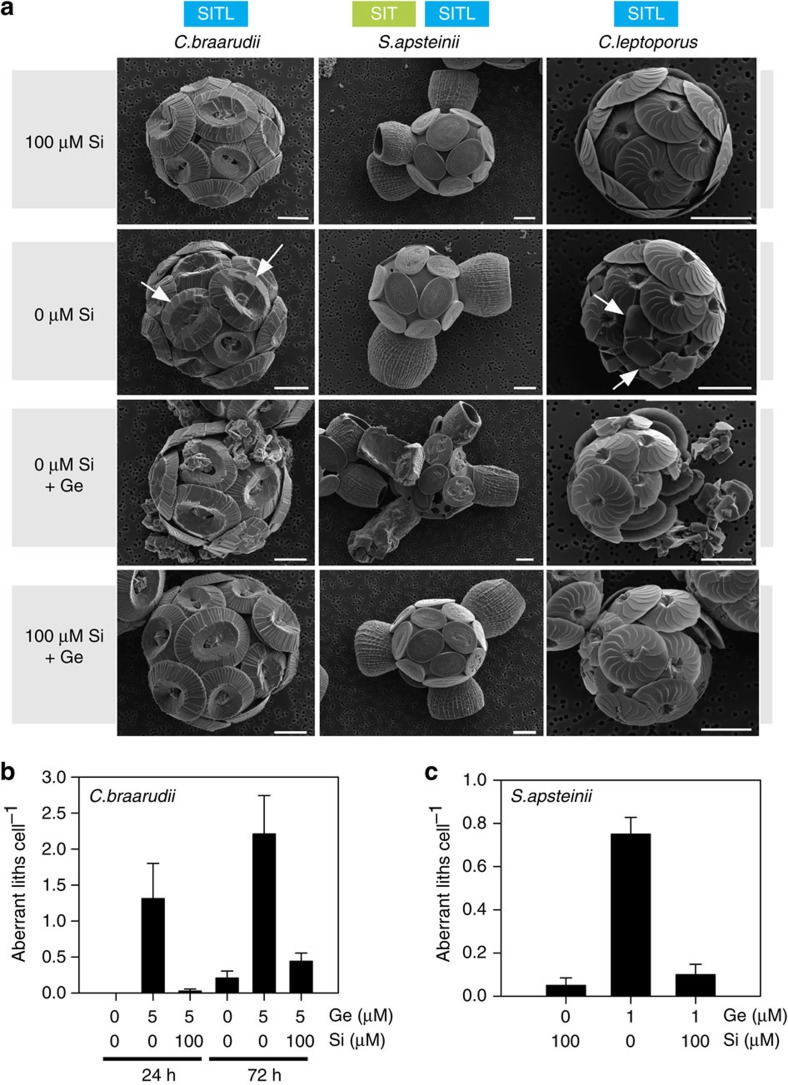
A role for Si in coccolith formation. (**a**) Representative SEM micrographs demonstrating the effects of Si limitation and Ge addition on coccolith production. *C. braarudii, S. apsteinii* and *C. leptoporus* were incubated for 72 h in very low Si seawater (<0.1 μM), which was amended with Ge (1 μM for *S. apsteinii* or 5 μM for the other two species). *C. braarudii* and *C. leptoporus* cells grown in very low Si appeared superficially similar to cells grown in Si-replete seawater (100 μM Si), but closer inspection revealed that many ‘blocky' coccoliths are apparent (arrowed), indicating a calcification defect related to the lack of Si. The addition of Ge resulted in the production of highly aberrant coccoliths in all three species. In *C. braarudii* these aberrant liths fail to integrate fully into the coccosphere and were often shed into the media. Both types of heterococcolith in *S. apsteinii* (the large cup-shaped lopadoliths and the small plate-like muroliths) exhibit extensive malformations. In *C. leptoporus* the aberrant coccoliths are all co-localized, suggesting that the newly formed liths in this species are secreted in a similar position in the coccosphere. The addition of 100 μM Si to Ge-treated cells markedly reduced the inhibitory effects on calcification. Scale bar, 5 μm. (**b**) Quantification of the production of aberrant coccoliths in *C. braarudii* grown in very low Si media for 24 and 72 h, amended with 5 μM Ge or 5 μM Ge+100 μM Si. For this experiment, only highly aberrant coccoliths were scored and more subtle coccolith malformations such as the ‘blocky' coccoliths observed under low Si were not scored. *n*=40 cells. For discarded liths 4–7 fields of view were scored containing at least 40 cells. The experiment was repeated four times and representative results are shown. (**c**) Quantification of the production of aberrant lopadoliths in *S. apsteinii* grown in low Si media for 72 h, amended with 1 μM Ge, or 1 μM Ge+100 μM Si. *n*=40 cells. The experiment was repeated four times and representative results are shown. Error bars denote s.e.

To test this hypothesis, we used the Si analogue germanium (Ge), which competitively inhibits Si uptake in diatoms^22^ and also prevents Si scale production in *P. neolepis* (Supplementary Fig. 7). In diatoms, Ge/Si ratios <0.01 do not have an inhibitory effect, but ratios >0.05 inhibit Si uptake and also disrupt Si metabolism within the cell^45^,^46^,^47^. Other silicifying algae, such as the chrysophytes *Synura petersenii* and *Paraphysomonas vestita*, are also sensitive to Ge, although growth in *Paraphysomonas* is only inhibited at much higher Ge/Si ratios than diatoms^48^,^49^. In contrast, non-silicified algae are reported to be largely unaffected by Ge^50^. Our initial experiments to screen for Ge sensitivity in coccolithophores were conducted in low-Si seawater (<0.1 μM), to ensure high Ge:Si ratios (>1). Observations with light microscopy and scanning electron microscopy (SEM) identified that coccolith formation in *S. apsteinii* was severely disrupted by the addition of 1 μM Ge, with 73% of cells displaying highly malformed coccoliths after 72 h (compared with 3.3% in Si-replete seawater; Fig. 3). The cup-shaped lopadoliths were severely misshapen, frequently exhibiting additional disorganized calcite precipitation at the apical rim, and the smaller disk-shaped muroliths also exhibited malformation. The addition of 5 μM Ge to *C. braarudii* and *C. leptoporus* resulted in the production of severely malformed coccoliths that failed to integrate into the coccosphere and were shed into the surrounding seawater (Fig. 3). In all three species, addition of 100 μM Si suppressed the disruptive effects of Ge on coccolith morphology, suggesting that Ge acts competitively with Si.

**Figure 4:**
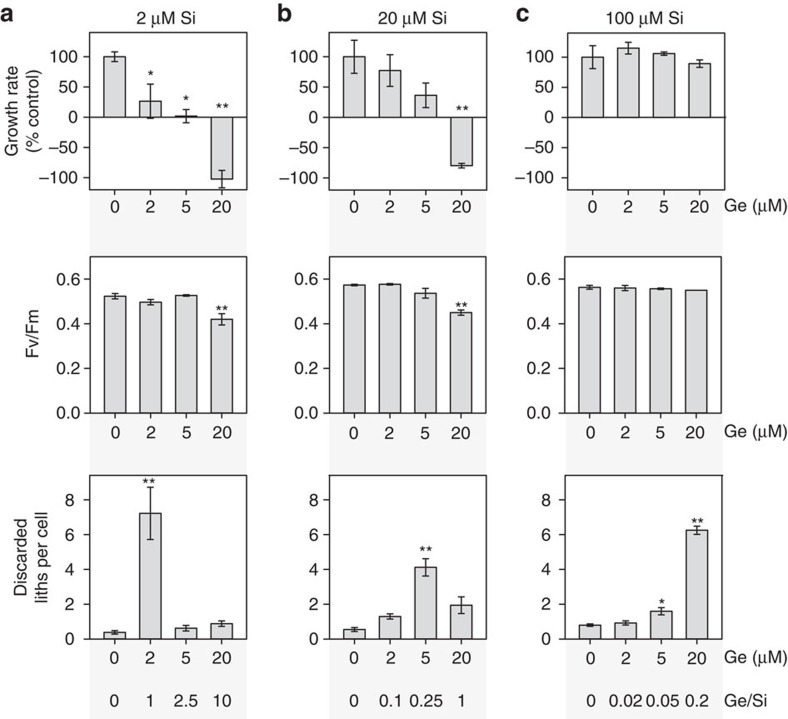
The inhibitory effects of Ge are dependent on the Ge/Si ratio. (**a**) *C. braarudii* cells were treated with 0, 2, 5 or 20 μM Ge for 48 h in seawater containing 2 μM Si. Effects on coccolith morphology were determined by counting the mean number of discarded liths relative to the cell density. Specific growth rate (per day) and photosynthetic efficiency (the quantum yield of photosystem II, *F*_v_/*F*_m_) were also determined. *P<0.05 and **P<0.01 denote treatments that differ significantly from the 0 μM Ge control (one-way ANOVA with Holm–Sidak *post hoc* test, *n*=3). Error bars denote standard errors. (**b**) *C. braarudii* cells treated as in **a** but in seawater containing 20 μM Si. (**c**) *C. braarudii* cells treated as in **a** but in seawater containing 100 μM Si. Ge had a much lower impact on coccolithophore physiology at higher Si concentrations, suggesting that Ge acts competitively with Si.

To examine the relationship between Ge and Si in greater detail, we grew *C. braarudii* cells at three different Si concentrations (2, 20 and 100 μM) and examined the effect of a range of Ge concentrations (0.5–20 μM Ge; Fig. 4). Because high Ge/Si ratios completely inhibit biosynthesis and growth in diatoms^46^, we also assessed the physiological status of the Ge-treated coccolithophores. We found that the inhibitory effects of Ge on calcification (assessed by the accumulation of discarded coccoliths in the media) are dependent on the ratio of Ge/Si, rather than the absolute concentration of Ge. For example, 2 μM Ge results in the production of many aberrant coccoliths at 2 μM Si, but its impacts at 20 and 100 μM Si are progressively reduced. The inhibitory effects of Ge on calcification in *C. leptoporus* and *S. apsteinii* were also dependent on the Ge/Si ratio (Supplementary Fig. 8). These data support the hypothesis that Ge is acting to competitively inhibit an aspect of Si uptake and/or metabolism that is required for production of coccoliths.

**Figure 5:**
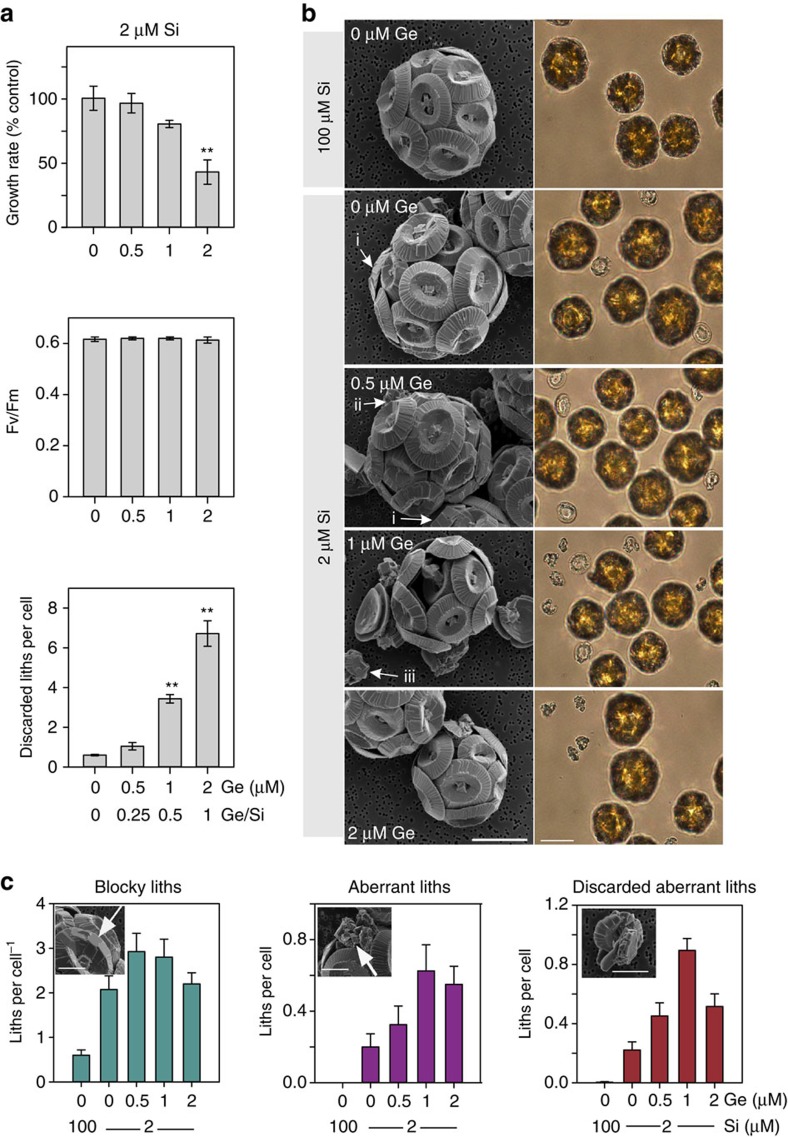
Ge causes defects in calcification at low Ge/Si ratios. (**a**) *C. braarudii* cells were treated with 0, 0.5, 1 or 2 μM Ge for 48 h in seawater containing 2 μM Si. Growth, photosynthetic efficiency and the number of discarded liths were determined. *P<0.05 and **P<0.01 denote treatments that differ significantly from the 0 μM Ge control (one-way ANOVA with Holm–Sidak *post hoc* test, *n*=3). (**b**) SEM (left panel) and bright-field microscopy (right panel) images of *C. braarudii* cells grown in Ge for 48 h (conditions described in **a**). Three classes of defective coccolith morphology were observed. (i) ‘Blocky' coccoliths where the overlapping arrangement of the distal shield is disrupted, but the shape of the coccolith is preserved. (ii) Aberrant coccoliths with highly disrupted morphology (iii) Discarded aberrant coccoliths that are not successfully integrated into the coccosphere. Note that even without Ge treatment ‘blocky' coccoliths can be observed at 2 μM Si, but these are not present at 100 μM Si. Scale bar, 10 μm. (**c**) Quantification of the defective coccolith morphology shown in **b**. At least 40 cells were scored for each treatment. For discarded liths four to seven fields of view were scored containing at least 40 cells. Error bars denote standard errors. Scale bar, 5 μm.

At high Ge/Si ratios (>1) both growth and calcification (accumulation of discarded coccoliths) were inhibited in *C. braarudii*. (Fig. 4). The maximum quantum yield of photosystem II (*F*_v_/*F*_m_) was only reduced at the very highest Ge/Si ratios. It is possible that the inhibition of growth results from the severe disruption of the calcification process. No effects on growth or photosynthetic efficiency were observed at low Ge/Si ratios, while coccolith defects were still observed, demonstrating that Ge had specifically disrupted calcification (Figs 4 and 5). The unique coccolith morphology of Ge-treated cells is distinct from defects in calcification caused by other stressors, such as nutrient limitation or high temperature^51^.

Detailed examination of Si-limited coccolithophores provided direct evidence for a requirement for Si in the calcification process. In Si-replete cultures, defects in coccolith morphology were almost completely absent (Figs 3 and 5). However, highly aberrant coccoliths were consistently observed at a low frequency in both *C. braarudii* and *C. leptoporus* cultures after transfer to very low Si seawater (without Ge) for 72 h (Fig. 3a,b). In addition to the appearance of highly aberrant coccoliths, many cells exhibited more subtle but significant defects in coccolith morphology due to Si limitation, such as disorganization of the overlapping elements of the distal shield (termed ‘blocky' morphology; Fig. 3a). Growth of *C. braarudii* was not inhibited after 8 days in very low Si (<0.1 μM; Supplementary Fig. 9), indicating that the defects in calcification are not caused by a general disruption of cellular physiology. Defective coccolith morphology was also apparent in *C. braarudii* and *C. leptoporus* cultures grown at 2 μM Si, compared to Si-replete cells grown at 100 μM Si (Fig. 5, Supplementary Fig. 8). This is an important observation as it shows calcification defects may occur at ecologically relevant Si concentrations. The slower growing *S. apsteinii* did not exhibit obvious defects in calcification after transfer to low Si for 72 h, but after 8 days clear defects in coccolith formation were observed, such as missing muroliths or incomplete lopadoliths (Supplementary Fig. 10).

**Figure 6:**
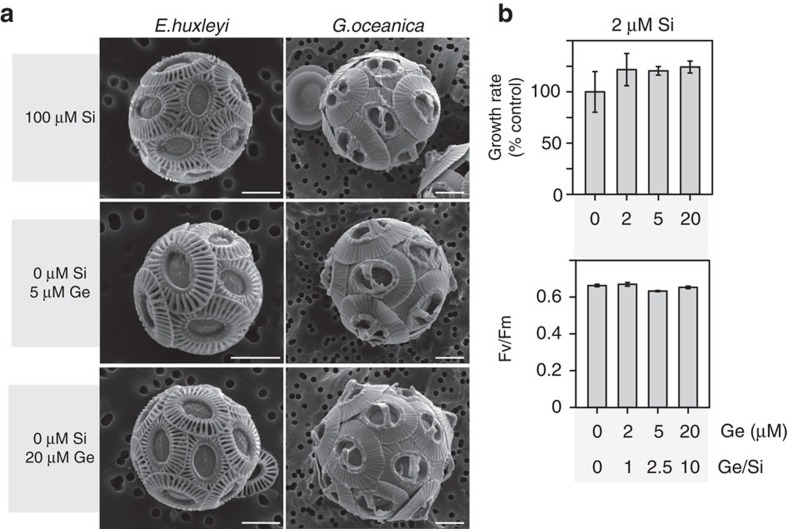
*Emiliania huxleyi* and *Gephyrocapsa oceanica* are insensitive to Ge. (**a**) Representative SEM micrographs for *E. huxleyi* and *G. oceanica* following treatment for 72 h in low Si (<0.1 μM) media with different additions of Ge (5 or 20 μM). No effect of Ge on coccolith morphology was observed in either species relative to the control grown in normal seawater media (100 μM Si). Si transporters (SIT/SITL) were not identified the genome of *E. huxleyi* or the transcriptome of *G. oceanica.* Scale bar, 2 μm. The results are representative of three independent experiments. (**b**) *E. huxleyi* cells were treated with 0, 0.5, 1 or 2 μM Ge for 48 h in seawater containing 2 μM Si. Mean specific growth rate and mean *F*_v_/*F*_m_ as a measure of photosynthetic efficiency were determined. No significant differences were noted between treatments and the 0 μM Ge control (one-way ANOVA, *n*=3). The results are representative of two independent experiments. Error bars denote s.e.

In combination, our results using Ge treatment and Si limitation strongly suggest that Si is required for calcification in certain coccolithophores. The dramatic effects of Ge on these species are surprising as most non-siliceous algae are considered to be insensitive to Ge^50^,^52^. However, many previous studies on coccolithophore physiology have focussed on *E. huxleyi*, a coccolithophore that lacks SITs or SITLs in its genome. When we examined the impact of Ge on *E. huxleyi* at very low Si (<0.1 μM Si), we found no effects on calcification, with normal coccospheres produced even in the presence of 20 μM Ge (Fig. 6a). Concentrations of Ge up to 20 μM also had no impact on the growth or photosynthetic efficiency of *E. huxleyi* at 2 μM Si (Fig. 6b), in clear contrast to the marked effects of Ge on *C. braarudii*. Furthermore, no Ge sensitivity was observed in two further coccolithophore species in which SITs or SITLs appear absent (from their available transcriptome sequence data); *G. oceanica*, a coccolithophore that is closely related to *E. huxleyi*, and *Pleurochrysis carterae* (Fig. 6a, Supplementary Fig. 11). Our results suggest that Si plays an important role in calcification in coccolithophores that possess SITs and/or SITLs, but this requirement for Si is not universal and is notably absent from the abundant bloom-forming coccolithophore species in modern oceans (the Noelaerhabdaceae)^4^.

## Discussion

While *P. neolepis* is the only known haptophyte exhibiting extensive silicification, our results point towards a much broader role for Si in haptophyte physiology. *P. neolepis* exhibits key similarities with other silicifying eukaryotes, but there is no evidence that silicification in this lineage arose from recent HGT. The mechanisms for silicification in *P. neolepis* have most likely been assembled independently from existing cellular components. Although *P. neolepis* contains a Si transporter belonging to the SIT family, the identification of a SIT in the coccolithophore *S. apsteinii* suggests that the presence of this family of Si transporters greatly predates the emergence of silicification in the haptophytes and may therefore have played an alternative role before being recruited for biomineralization. In diatoms, Thamatrakoln and Hildebrand^22^ have proposed that SITs may have played an ancestral role in preventing excessive accumulation of intracellular Si in the Si-rich waters of Mesozoic oceans, before they were recruited for frustule formation.

SITs exhibit a very limited distribution in eukaryotes (stramenopiles, haptophytes and choanoflagellates). In the absence of evidence for HGT, an alternative explanation is that this distribution results from multiple losses of a gene that was present in the last common ancestor of these lineages. However, as this ancestor was most likely close to the last common ancestor of all eukaryotes, this scenario requires gene loss of SITs on a massive scale. Two factors that may have contributed to the loss of SITs in eukaryotes are the potential functional redundancy between SITs and SITLs and the extensive depletion of Si from surface oceans in the Cenozoic. However, an alternative scenario that does not require such extensive gene loss is possible as the phylogenetic position of the haptophytes is not fully resolved. Recent phylogenomic evidence suggests a specific association between haptophytes and stramenopiles^53^,^54^, with Stiller *et al*.^54^ proposing that haptophytes acquired their plastids following endosymbiosis of a photosynthetic stramenopile belonging to the ochrophyta (which includes diatoms and chrysophytes). The associated endosymbiotic gene transfer therefore provides a mechanism through which the haptophytes may have acquired SITs from stramenopiles. The phylogeny of the SITs is not at odds with this scenario, as the proposed endosymbiosis would have occurred before the extensive radiation of the stramenopiles and the haptophytes, but it does infer that the SITs have been lost extensively in both of these taxonomic groups. This scenario does not explain the presence of SITs in choanoflagellates. Although HGT of SITs to or from choanoflagellates is not supported by the phylogeny, it cannot be ruled out and there is evidence for extensive HGT from algae into choanoflagellates^55^.

Clearly, there are broader evolutionary questions relating to the phylogeny of the haptophytes that must be addressed before we can fully determine the origins of the SITs. Further understanding of the function and roles of the SITLs may also provide important insight into these processes. Nevertheless, our results clearly suggest that an expanded family of SITs were present in ancestral haptophytes and that both SITs and SITLs were present in the ancestor of the calcifying coccolithophores. Therefore, it seems likely that this ancestor possessed the capacity for Si uptake. As Si exhibits the ability to modulate calcite precipitation *in vitro*^42^,^43^, its presence in ancestral coccolithophores may even have facilitated the emergence of extensively calcified coccoliths. We have provided evidence of a role for Si in coccolith formation in *S. apsteinii, C. braarudii* and *C. leptoporus*. These results identify that Si uptake via SITs is an important common mechanism contributing to very different modes of biomineralization in two of the major phytoplankton lineages, the diatoms and the coccolithophores.

Lsi2 was not found in coccolithophores with SITs and SITLs, suggesting that its cellular role in *P. neolepis* and diatoms may relate to the process of silicification. In plants, Lsi2 is proposed to act as a H^+^/silicic acid exchanger, using an inward H^+^ gradient to drive the efflux of silicic acid across the plasma membrane^28^. In silicifying organisms, H^+^/silicic acid exchangers could act to load the acidic silica deposition vesicle, using the H^+^ gradient across the vesicle membrane to drive the accumulation of silicic acid. It will therefore be important to identify the cellular localization of Lsi2 in *P. neolepis* and diatoms.

We do not yet know the cellular mechanisms through which Si contributes to the calcification process. Previous workers have identified a role for Si in bone formation in vertebrates^56^,^57^. However, the primary role of Si in bone formation appears to relate to the synthesis of collagen to form the underlying organic matrix, rather than a direct role in the mineralization process^58^,^59^. More recently, it has been demonstrated that silica plays an important role in formation of cystoliths, small calcium carbonate deposits that are found in the leaves of some land plants^41^,^60^. Although silica is only a minor component of cystoliths, it is essential for the formation of the amorphous calcium carbonate phase that comprises the bulk of the structure^60^. These studies suggest that Si could act to modulate coccolith formation through a number of mechanisms. Further elucidation of its precise role will enable important insight into the cellular mechanisms of calcification in coccolithophores, which remain poorly understood.

Significantly, our results suggest that requirement for Si in coccolithophore calcification may have been lost by the Noelaerhabdaceae and Pleurochrysidaceae. There are other potential evolutionary scenarios that we cannot rule out at this stage, such as independent evolution of the Si requirement within the Zygodiscales and the Coccolithales, although these scenarios are less parsimonious. The marked decline of surface ocean silicate in the Cenozoic also suggests that loss of the requirement for Si would be more likely than gain. These evolutionary events have important implications for coccolithophore ecology and prompt a re-evaluation of the widely held view that the coccolithophores do not require Si. The Si-requiring coccolithophores identified in this study are important marine calcifiers, with *C. braarudii* and *C. leptoporus* contributing significantly to calcite flux to the deep ocean in large parts of the Atlantic Ocean^5^,^61^. Although the requirement of these coccolithophores for Si is likely to be considerably lower than that of extensively silicified organisms, Si limitation clearly impairs their ability to calcify. Whether these species encounter significant Si limitation in natural seawaters and can compete effectively for this resource with diatoms and other silicified plankton must be resolved. However, concentrations of silicate in the surface ocean can often reach very low levels, particularly after diatom blooms^62^. It is possible that small fast-growing coccolithophores, which are best suited to exploit the nutrient-depleted waters following a diatom bloom, may have encountered selective pressure to uncouple calcification from Si uptake to avoid Si limitation. The bloom-forming coccolithophores belonging to the Noelaerhabdaceae, such as *E. huxleyi* and *G. oceanica*, may therefore have developed alternative cellular mechanisms to replace the role of Si in coccolith formation. The Noelaerhabdaceae are the most abundant and broadly distributed coccolithophores in modern oceans and their ability to form extensive blooms (often in Si-depleted waters) has likely contributed to their considerable ecological success^16^,^17^,^18^. The differing requirements for Si may therefore have had a profound impact on the physiology of modern coccolithophores and contributed significantly to the evolution and global distribution of this important calcifying lineage.

## Methods

### Algal strains and culture growth

*Prymnesium neolepis* (NCBI Tax ID 284051) strains TMR5 (RCC3432—Sea of Japan) and PZ241 (RCC1453—Mediterranean Sea) were obtained from the Roscoff Culture Collection. Strain TMR5 was used for all physiological analyses and for RT–PCR. Strain PZ241 was used to generate the transcriptome. Cultures of *P. neolepis* were maintained in filtered seawater (FSW) supplemented with f/2 nutrients (including 100 μM Na_2_SiO_3_.5H_2_O) under irradiance of 80–100 μmol s^−1^ m^−2^ (18:6 h light:dark) at 18 °C. Stock cultures of the coccolithophores *Coccolithus braarudii* (formerly *Coccolithus pelagicus* ssp *braarudii)* (PLY182G), *Emiliania huxleyi* (PLY-B92/11) and *Pleurochrysis carterae* (PLY406) were maintained in FSW supplemented with f/2 nutrients (without added Si) and Guillard's vitamins as previously described^40^. *Calcidiscus leptoporus* (RCC1130), *Gephyrocapsa oceanica* (RCC1303) and *Scyphosphaera apsteinii* (RCC1456) were maintained in f/2 supplemented with 10% K medium. All coccolithophore cultures were grown at 15 °C under 80–100 μmol s^−1^ m^−2^ irradiance (14:10 h light:dark).

### Manipulation of seawater Si and addition of Ge

To examine the effect of Si and Ge on coccolithophores, we used a batch of seawater from the Western English Channel in which Si was naturally low (measured at 2 μM using the molybdate-ascorbate assay^63^). This batch of seawater was used for all subsequent analyses involving the effect of Ge on coccolithophores, except where very low Si concentrations were required (see below). Si concentration was amended by the addition of Na_2_SiO_3_.5H_2_O. Ge was added in the form of GeO_2_, to give concentrations ranging from 0.5–20 μM. f/2 nutrients (without Si) were added and all coccolithophore cultures were grown under identical conditions (15 °C under 80–100 μmol s^−1^ m^−2^ irradiance, 14:10 h light:dark). For growth experiments, coccolithophore cultures were acclimated to 2 μM Si for several generations (1–2 weeks) before the onset of the experimental period. For SEM analysis, all cultures were maintained in 100 μM Si for 1–2 weeks before the onset of the experimental period to prevent accumulation of aberrant coccoliths in the control.

Very low Si seawater was prepared using diatoms to deplete Si as described previously^64^. One-litre batches of f/2 FSW (without added Si) were inoculated with the diatom *Thalassiosira weissflogii* and allowed to grow into stationary phase (6–10 days). Diatoms were removed by two passages through 0.2-μm filters and the Si concentration was verified on an autoanalyser (Bran+Luebb, Germany) using a molybdate-ascorbate assay^63^. The initial Si in Gulf Stream Seawater was 5.4 μM and after diatom depletion the Si was below the level of detection (<0.1 μM, hereto referred to as 0Si f/2 FSW). Before inoculation of treatment media, aliquots of cells were washed at least twice by allowing them to settle, drawing off the overlying media, and resuspending in 0Si f/2 FSW. An inoculum of 0Si f/2 FSW washed cells was then added to a tube of the treatment media and monitored over 72 h. Care was taken to ensure final cell numbers did not exceed 2 × 10^4^ cells per ml for *E. huxleyi*, the most rapidly growing of the three species, thus avoiding any significant changes to the carbonate chemistry of the culture medium over the course of the experimental incubations.

### Physiological measurements

Growth rates of coccolithophore cultures were determined by cell counts using a Sedgewick-Rafter counting chamber (*C. braarudii, P. carterae*) or a Neubauer improved haemocytometer (*E. huxleyi).* Specific growth rates (per day) were determined from the initial and final cell densities (*N*_t0_, *N*_t1_) using the formula *μ*=(ln(*N*_t1_)−ln(*N*_t0_))/*t*). For Ge-treated *C. braarudii* cultures, an initial cell density of 1.2 × 10^4^ cells per ml was used to ensure sufficient biomass was available after 48 h for measurements of chlorophyll fluorimetry. For these short-term incubations, the control cultures exhibited a specific growth rate between 0.24–0.35 per day and growth of the Ge-treated cultures is shown as a percentage of the control. For Si-limited cultures, an initial cell density of 4.5 × 10^3^ cells per ml was used and growth was monitored over 8 days. Discarded coccoliths of *C. braarudii* were also counted for selected experiments. As coccolith morphology can be difficult to determine accurately by light microscopy, we did not discriminate between intact and aberrant liths in these analyses. To assess the performance of the photosynthetic apparatus, the maximum quantum yield of photosystem II was determined using a Z985 AquaPen chlorophyll fluorimeter (Qubit Systems, Kingston, Canada). Statistical analyses of these data were performed in SigmaPlot v12.0 software (Systat Software Inc, London, UK).

### Fluorescence microscopy of *P. neolepis* silica scales

One millilitre of *P. neolepis* cells was incubated with the fluorescent dye LysoTracker yellow HCK-123 or LysoSensor Yellow/Blue DND-160 (Invitrogen; 1 μM, 10 h). Fluorescently labelled scales were imaged by confocal laser scanning microscopy (Zeiss LSM 510 microscope). HCK-123 was viewed using excitation at 488 nm and emission at 500–550 nm. DND-160 was viewed using multiphoton excitation at 740 nm with emission at 435–485 nm and 500–550 nm. Chlorophyll autofluorescence was also detected (emission 650–710 nm).

### Extraction of silica-associated organic components

Organic components were extracted from the silica scales of *P. neolepis* using a modified protocol for diatom frustules^65^. Cells in mid-exponential growth phase were harvested by low pressure filtration and pelleted by centrifugation (500 × *g*, 5 min, Thermo Scientific, Waltham, MA). The cells were disrupted by the addition of 10 ml of lysis buffer (2% SDS, 100 mM EDTA, 0.1 M Tris pH 8.0), vortexed and centrifuged at 6,000*g* for 10 min. The pellet containing the silica scales was washed with lysis buffer a further five times to remove cellular organic material. The silica scales were further purified by centrifugation through a 50% glycerol cushion (3,200*g*, 2 min) to remove any traces of contaminating low-density organic material, such as the smaller organic scales. The purity of the silica scale preparation was assessed by light microscopy (Nikon Ti Eclipse, Tokyo, Japan) and electron microscopy (both SEM and transmission electron microscopy). No contamination with cell debris or organic scales was observed in the purified preparations of silica scales, although organic scales could clearly be viewed in crude cell extracts. To dissolve the silica component of scales, 2 ml of 10 M NH_4_F was added to 30–100 mg biosilica sample and vortexed until the pellet was dissolved. 0.5 ml of 6 M HCl was then added to the mixture, vortexed, and the pH was adjusted to 4.5 with 6 M HCl. The sample was incubated at room temperature for 30 min before centrifugation (3,200*g*, 15 min) and the supernatant was transferred to a 3 kDa cut-off filtration column (Amicon) to concentrate and desalt protein. The concentrate was washed sequentially with 5 ml of 500 mM ammonium acetate, 5 ml of 200 mM ammonium acetate and three times with 5 ml of 50 mM ammonium acetate. The sample was then further concentrated to 150–400 μl and analysed using Tricine/SDS–PAGE with Coomassie Blue staining to stain both proteins and LCPAs^66^. Staining with silver stain or Stains-All (Sigma), which do not bind to LCPAs, was used to verify that the lower molecular weight component did not contain protein. A trypsin digest was also conducted, where 10 μl of the NH_4_F soluble extract was incubated with 2 μg of TPCK (tosyl phenylalanyl chloromethyl ketone)-treated trypsin in 100 mM Tris-HCl at pH 8.8 at 37 °C (18 h). Analysis by Tricine/SDS–PAGE revealed that the higher molecular weight component had been removed by trypsin, but the low-molecular-weight component (LCPA) remained. To further confirm that silica scales were not contaminated with cellular debris or organic scales, the purified silica scales were extracted with 5 ml buffer (100 mM EDTA, 0.1 M Tris pH 8.0) in the absence of NH_4_F dissolution. No organic components were observed following Tricine/SDS–PAGE analysis, indicating that the organic components observed following treatment with NH_4_F are released by silica dissolution.

### Protein identification from silica scale extract

Following Tricine/SDS–PAGE, protein bands were excised from the gel and analysed by peptide mass fingerprinting using a tryptic digest (Alta Bioscience, Abingdon, UK). The *P. neolepis* transcriptome was used to create a reference proteome. A single protein (LPCL1) was identified, with 8–16 unique peptides identified in each sample. The protein identification was repeated at an alternative facility (Mass Spectrometry Facility, Biosciences, University of Exeter, UK) using an independent protein extract. This gave an identical result identifying 8 unique peptides for LPCL1.

### LCPA purification from silica scale extract

LCPAs were separated from the protein fraction by ultrafiltration of 500 μl of the NH_4_F soluble extract through a 10 kD MW filtre. The LCPAs were then further purified by cation exchange through 2 ml of high S strong cation exchange resin (Bio-Rad, Hemel Hempstead, UK). The column was prepared by washing sequentially with 10 ml of deionised water, 10 ml of 2 M ammonium acetate and then three further times with deionised water. The NH_4_F extraction was diluted (4.5:100) with deionised water and passed through the column. The resin was then washed three times with 1 ml of 200 mM ammonium acetate and polyamines were eluted by 4 sequential additions of 1 ml of 2 M ammonium acetate. The eluant was neutralized with acetic acid and lyophilized. Long-chain polyamines were analysed by electrospray ionization mass spectrometry (ESI-MS) using an amaZon speed mass spectrometer (Bruker, Bremen). Samples were diluted in H_2_O/CH_3_CN (50/50), and injected by direct infusion at a flow rate of 500 nl min^−1^ using a Captive Spray ion source. MS and MSn spectra were acquired in positive ion mode.

### Generation of the *P. neolepis* transcriptome

A 100-ml culture of *P. neolepis* strain PZ241 growing in standard conditions (mid-exponential phase, f/2+Si, other growth conditions as described above) was used to generate the transcriptome. Cells were collected 4 h into the light cycle by centrifugation (500*g*, 5 min). RNA was extracted using the Trizol method (Invitrogen, Paisley, UK), with additional purification using an RNeasy kit (Qiagen, Venlo, Netherlands). Following reverse transcription using oligo-dT primers, *P. neolepis* complementary DNA was sequenced by Illumina technology, generating 64,548,084 paired end reads of 75 bp (Genoscope, Evry, France). The paired end reads were assembled by Trinity^67^, producing 118,473 transcripts, including alternative forms of a total of 83,175 transcripts.

### Reverse transcription PCR

Reverse transcription PCR (RT–PCR) was used to verify the expression of selected genes (*LPCL1*, *SIT* and *SITL)* identified in the haptophyte transcriptomes. Fifty-millilitre cultures of *P. neolepis* (TMR5), *C. braarudii, C. leptoporus* and *S. apsteinii* were grown in standard conditions (as described above). Cells were collected ∼4 h into the light cycle by centrifugation (500*g*, 5 min). RNA was extracted using the Trizol method (Invitrogen), with additional purification using an RNeasy kit (Qiagen). Complementary DNA was synthesized using either oligo-dT primers (*PnSIT1*) or gene specific primers (all other products) using Superscript III reverse transcriptase (Invitrogen). Gene products were then amplified by PCR (95 °C for 30 s, 54 °C for 30 s, 72 °C for 60 s, 35 cycles) (Supplementary Table 3). PCR products were sequenced to confirm the amino-acid sequence of the predicted protein product (Source BioScience, Cambridge, UK). The nucleotide sequences of *P. neolepis* SIT1 and LPCL1 obtained from the TMR5 strain were 100% identical at the nucleotide level to those identified in the PZ241 transcriptome.

### Bioinformatic analyses

Known proteins associated with silicification from diatoms, sponges and land plants were used to search the haptophyte transcriptomes (Supplementary Table S1). The additional haptophyte transcriptomes were obtained from the Marine Microbial Eukaryote Sequencing Project (MMETSP; http://camera.calit2.net/mmetsp/)^35^. The genomes of *Emiliania huxleyi* v1.0 and *Thalassiosira pseudonana* v3.0 were obtained from the Joint Genome Institute (JGI; http://genome.jgi.doe.gov/). Further searches were performed at NCBI (http://blast.ncbi.nlm.nih.gov/Blast.cgi), including Transcriptome Shotgun Assembly (TSA) and Expressed Sequence Tag (EST) databases. Databases were searched using BLASTP and TBLASTN. Position-specific iterative BLAST (PSI-BLAST) was used to identify highly conserved motifs in proteins that exhibit low levels of sequence identity (for example, lipocalins). Each potential hit was manually inspected using a multiple sequence alignment to identify conserved residues and then phylogenetic analyses were performed using both neighbour-joining and maximum likelihood methods within the MEGA5 software package to assess the relationship with known proteins^68^. For detailed phylogenetic analysis of SITs and SITLs, multiple sequence alignments were generated using MUSCLE and manually inspected for alignment quality. After manual refinement, GBLOCKS 0.91b was used to remove poorly aligned residues^69^ and then ProtTest was used to determine the best substitution model (WAG with gamma and invariant). Maximum likelihood phylogenetic trees were generated using PhyML3.0 software with 100 bootstraps. Bayesian posterior probabilities were calculated using BEAST v1.8, running for 10,000,000 generations^70^. The identification of potential transmembrane domains in SITLs was performed using Phobius and TMHMM.

### Electron microscopy

SEM images of *P. neolepis* scales were acquired with a JEOL 5000 and JEOL 7001 F microscopes (Jeol, Japan) at 15 keV accelerating voltage. Scales were collected using lysis buffer (2% SDS, 100 mM EDTA, 0.1 M Tris pH 8.00) as described above, but were additionally cleaned by heating at 95 °C for 10 min in the lysis buffer. Purified *P. neolepis* silica scale material was dried and sputter coated with gold or chromium before imaging. Samples of coccolithophores for SEM were collected by filtration onto a 13-mm 0.4-μm Isopore filter (Millpore EMD), followed by a rinse with 10 ml of 1 mM HEPES buffer (pH 8.2) to remove salts. Filters were air-dried, mounted onto an aluminium stub and sputter coated with 10 nm Pt/Pd (Cressington, USA). Samples were examined with a Phillips XL30S FEG SEM (FEI-Phillips, USA) and imaged in high-resolution secondary electron mode with beam acceleration of 5 kV. Three categories of coccolith morphology were scored. (i) ‘Blocky' coccoliths where the overlapping arrangement of the distal shield is disrupted, but the overall shape of the coccolith is not disrupted (*C. braarudii* and *C. leptoporus* only). (ii) ‘Aberrant' coccoliths were classified as coccoliths that clearly departed from the typical morphology for any given species. (iii) ‘Discarded aberrant' coccoliths were classed as those aberrant coccoliths that failed to integrate into the coccosphere. To analyse coccolith morphology, at least 40 cells per treatment were scored for the number of malformed coccoliths present in the coccosphere. Coccoliths on the underside of cells could not be scored and the resultant underestimate of coccoliths per cell was assumed to be the same for any given species. Discarded aberrant coccoliths were counted in four to seven random fields of view in which both cells and loose aberrant coccoliths on the filter were scored (at least 40 cells in total were scored for each treatment). Ge-treated cultures for SEM analysis were grown in single replicates. Each experiment was repeated on multiple independent occasions and in each case the effects of Ge were highly reproducible. A representative example of each experiment is shown. Error bars denote standard error.

## Additional information

**Accession codes:** Sequences from and were submitted to GenBank (KP793098-KP793099, KR677451).

**How to cite this article:** Durak, G. M. *et al*. A role for diatom-like silicon transporters in calcifying coccolithophores. *Nat. Commun.* 7:10543 doi: 10.1038/ncomms10543 (2016).

## Supplementary Material

Supplementary Information Supplementary Figures 1-11 and Supplementary Tables 1-3.
