# Supplementary material for: A role for diatom-like silicon transporters in calcifying coccolithophores
Source: Nat Commun. 2016 Feb 4;7:10543. doi: 10.1038/ncomms10543 (PMC4742977; doi:10.1038/ncomms10543)
Supplement: Supplementary Information — Supplementary Figures 1-11 and Supplementary Tables 1-3. [file ncomms10543-s1.pdf]

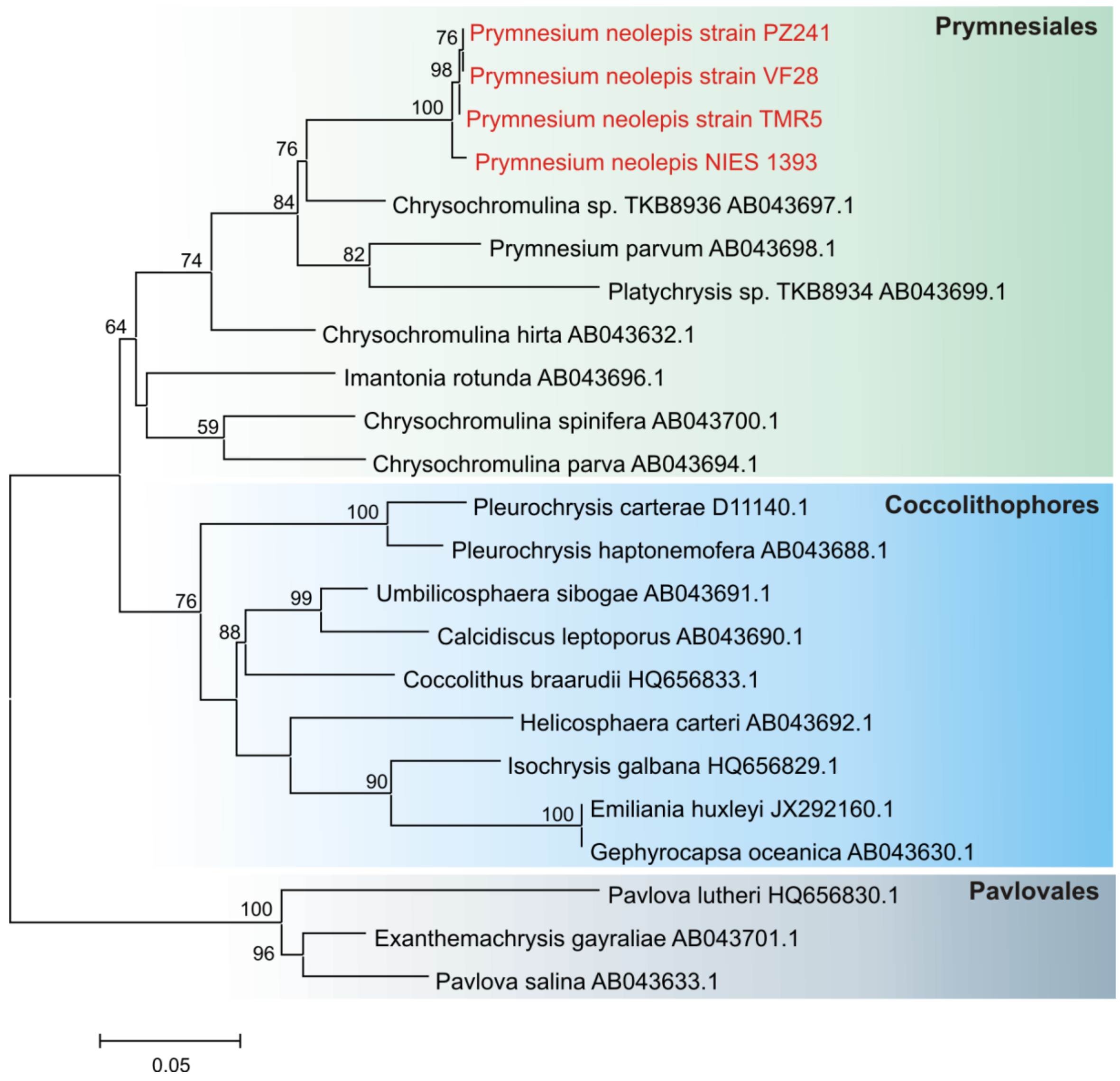

Supplementary Figure 1: Phylogenetic tree of *rbcL* from haptophytes

A maximum likelihood phylogenetic tree of *rbcL* sequences from haptophytes is shown, demonstrating the relationship between different *P. neolepis* isolates. *P. neolepis* strain TMR5 was used for all experimental analysis. Strain PZ241 was used to generate the transcriptome. The tree was generated using a nucleotide alignment for *rbcL* (final alignment size 673 bases). Numbers above nodes represent bootstrap values >70% (1000 bootstraps were performed). Scale bar represents nucleotide substitutions per site.

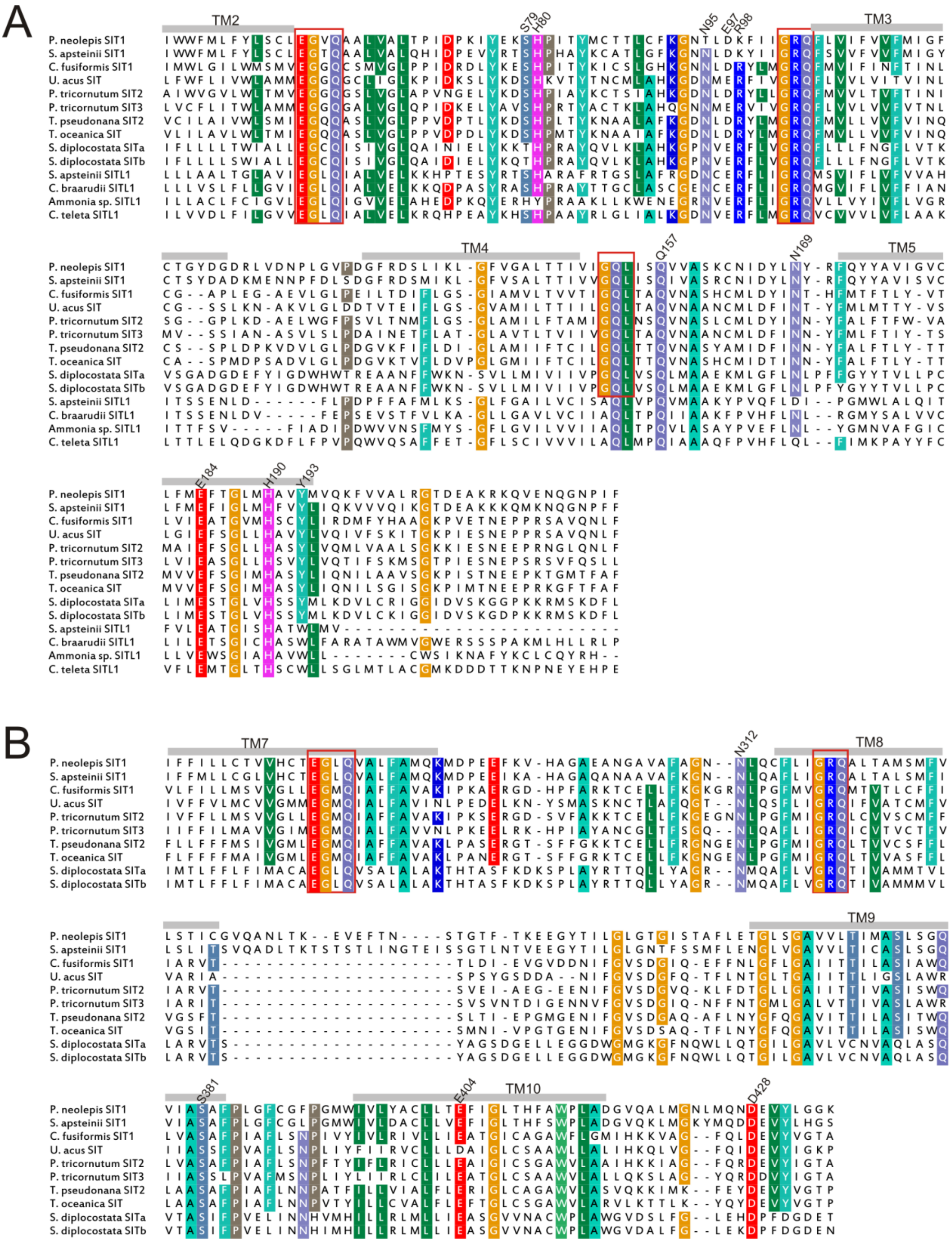

Supplementary Figure 2: Multiple sequence alignment of the SIT family of Na^{+}-coupled Si transporters

A) Multiple sequence alignment showing N-terminal region of SITs aligned to SITL sequences. An initial alignment of 35 SITs from diatoms, chrysophytes, choanoflagellates and haptophytes was analysed to identify amino acid residues that were highly conserved across SITs from diverse evolutionary origins. For simplicity, only 10 SITs are displayed in alignment shown in the figure. The highly conserved EGxQ, GRQ and GQL motifs (boxed) are found in all SITs. The positions of several other highly conserved charged or polar amino acids are also indicated. In some instances the conserved residue is replaced with an amino acid with similar properties (e.g. R98 to lysine in haptophytes) is present. The numbers indicate the relative position of each amino acid in *C. fusiformis* SIT1. The predicted positions of the transmembrane domains (TM) in CfSIT1 are shown. The alignment also shows the SITLs aligned to the N-terminal 5-TM domain of SITs. The SITLs contain the EGxQ and GRQ motifs but G is replaced by A in the GQL motif. Many of the highly conserved residues identified in SITs are also conserved in SITLs. The alignment represents amino acid residues 47-221 of *C. fusiformis* SIT1. Accession numbers: *U. acus* AFS18271.1; *C. fusiformis* AAC49653.1; *P. tricornutum* SIT2 ACJ65493.1, SIT3 ACJ65494.1; *T. pseudonana* ABB81826.1; *T. oceanica* EJK72537.1; *S. diplocostata* SITa HE981735.1, SITb HE981736.1. B) Multiple sequence alignment showing the C-terminal region of SITs. The alignment represents amino acid residues 262-435 of *C. fusiformis* SIT1. All other details as listed above for (A).

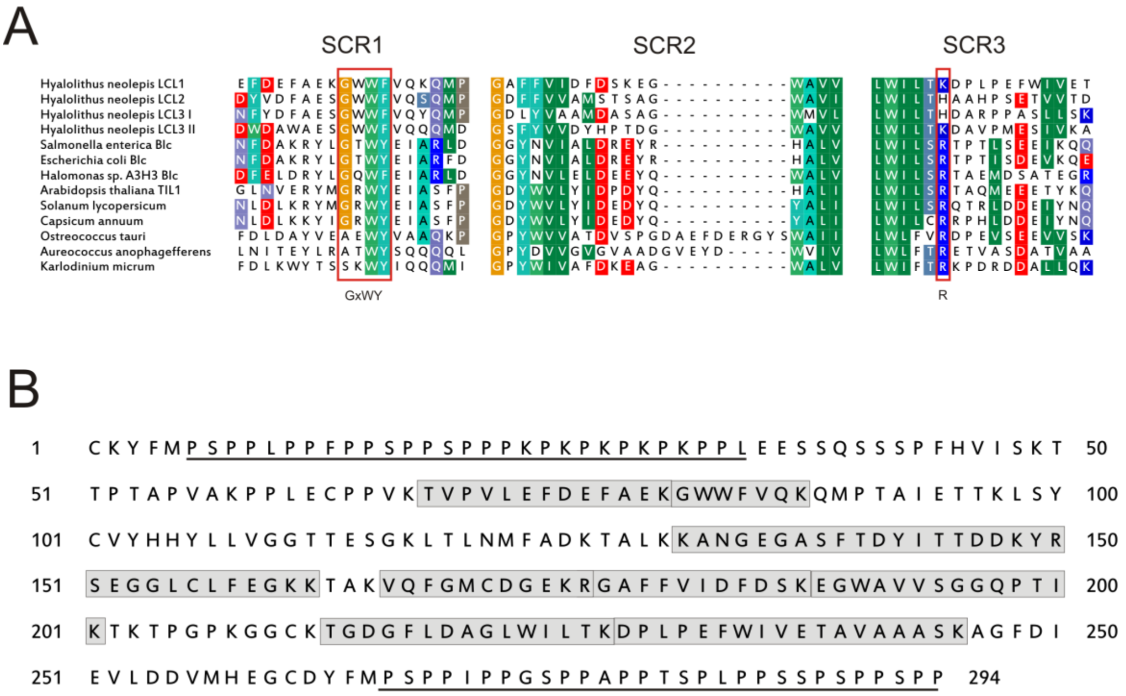

Supplementary Figure 3: Identification of a lipocalin-like protein from *P. neolepis*

A) A multiple sequence alignment of lipocalins. The *P. neolepis* lipocalin-like protein (LPCL1) identified following NH_4F-mediated dissolution of the silica scales is shown aligned to bacterial lipocalins and temperature-sensitive lipocalins from plants. Two further predicted proteins (LPCL2 and LPCL3) were found in the *P. neolepis* transcriptome exhibiting a high similarity to LPCL1, although these were not identified in the silica scale proteome. Both LPCL2 and LPCL3 exhibit the proline/lysine-rich flanking regions of LPCL1, although LPCL3 exhibits tandem lipocalin domains. Lipocalins are not well-conserved at the sequence level, but exhibit a highly conserved structure. Three structurally conserved regions (SCR) found in all lipocalins are also found in the *P. neolepis* proteins. The GxWY motif in SCR1 is largely conserved (GWWF), although the conserved arginine in SCR3 has been replaced by either a lysine or a histidine. LPCL1 exhibits weak similarity to lipocalin-like proteins from marine algae, but these lack the proline-rich flanking regions. Accession numbers: *S. enterica* WP_023185648.1; *E. coli* WP_021530789.1; *H.* A3H3 WP_022522704.1; *A. thaliana*

NP_200615.1; *S. lycopersicum* NP_001234515.1; *C. annuum* AAT71313.1; *O. tauri* XP_003083465.1; *A. anophagefferens* ABV22241.1; *K. micrum* ABV22241.1. **B)** The putative amino acid sequence of LPCL1. Grey boxes indicate the peptides identified by mass spectrometry. The proline/lysine-rich flanking regions are underlined. The predicted size of the *LPCL1* product (31.6 kD) differs from the size predicted by gel electrophoresis (50 kDa), which may be due to an incomplete sequencing of the cDNA product or the result of post-translational modifications affecting gel mobility, as demonstrated in the silaffins from diatom frustules.

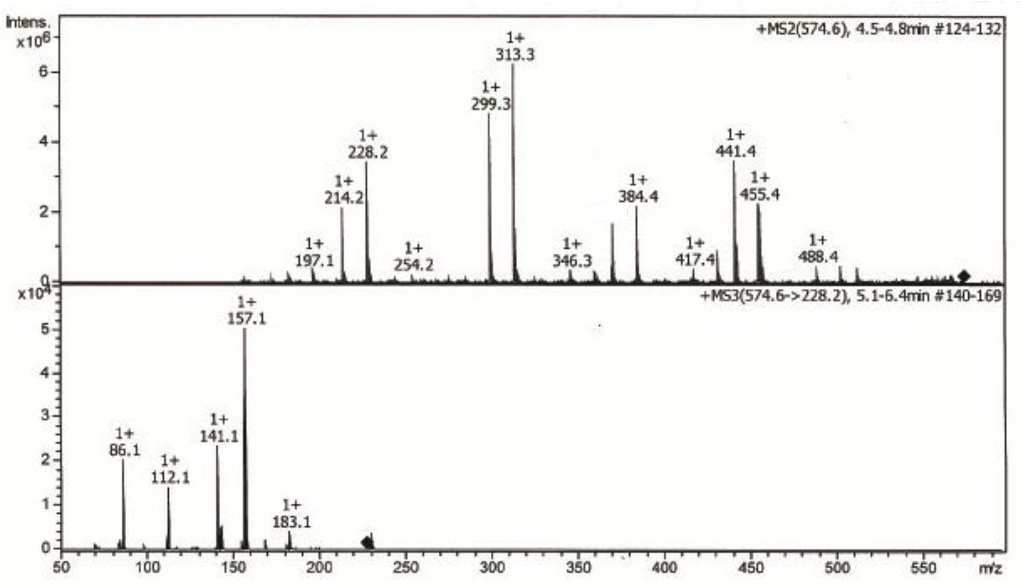

Supplementary Figure 4: Tandem mass spectometry of *P. neolepis* LCPAs

Tandem mass spectometry of the [m+H$^+$] 573 fragment from *P. neolepis.* The mass spectra of *P. neolepis* LCPAs revealed a 71 Da fragmentation pattern characteristic of other LCPAs from diatoms and sponges. However, the mass peaks themselves were identical to LCPA-modified lysine residues found in to diatom silaffins. MS/MS of the [m+H$^+$] 573 ion from *P. neolepis* reveals a highly similar fragmentation pattern to the [m+H$^+$] 573 ion obtained from diatom silaffins.

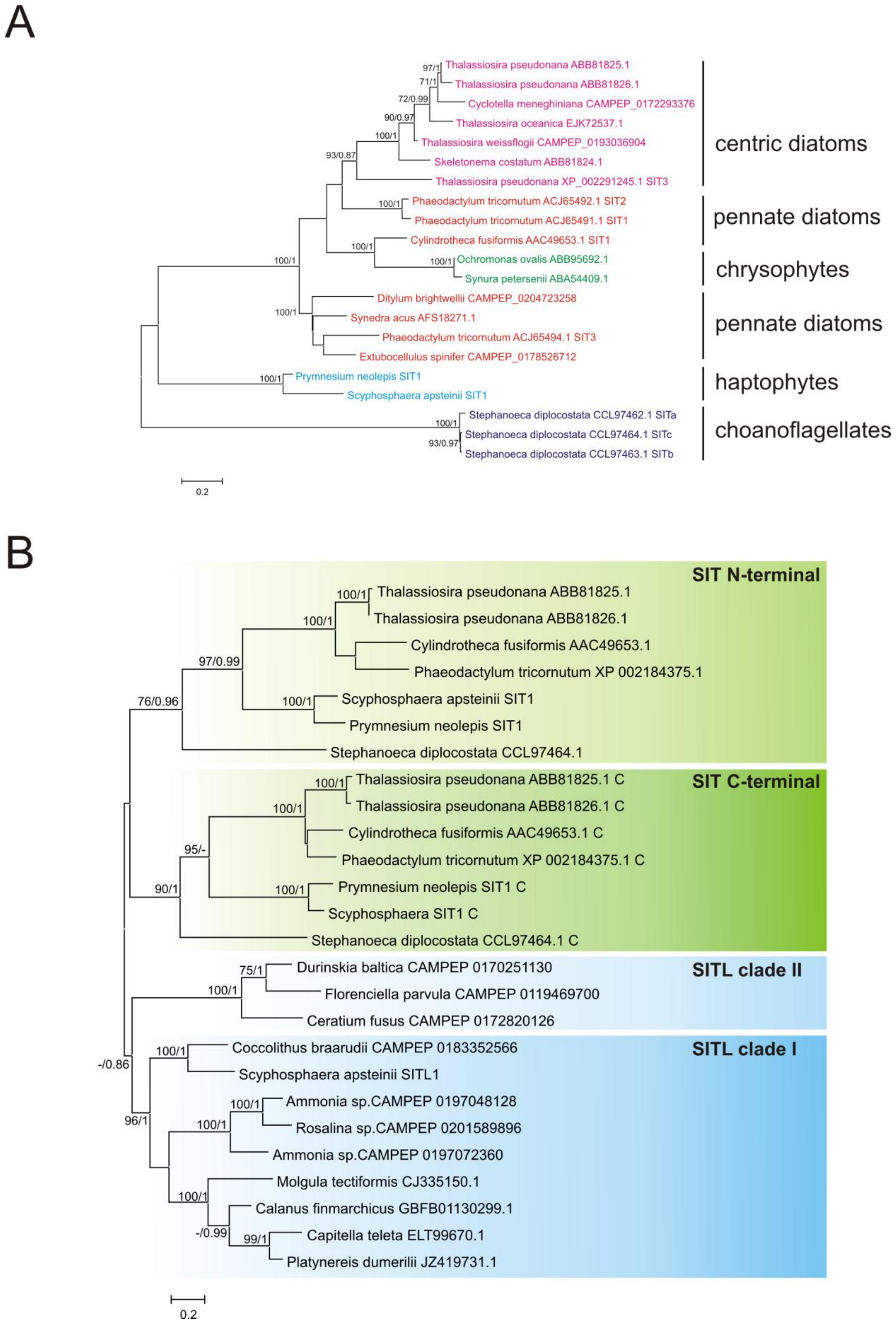
A
Thalassiosira pseudonana ABB81825.1
Thalassiosira pseudonana ABB81826.1
Cyclotella meneghiniana CAMPEP_0172293376
Thalassiosira oceanica EJK72537.1
Thalassiosira weissflogii CAMPEP_0193036904
Skeletonema costatum ABB81824.1
Thalassiosira pseudonana XP_002291245.1 SIT3
Phaeodactylum tricornutum ACJ65492.1 SIT2
Phaeodactylum tricornutum ACJ65491.1 SIT1
Cylindrotheca fusiformis AAC49653.1 SIT1
Ochromonas ovalis ABB95692.1
Synura petersenii ABA54409.1
Ditylum brightwellii CAMPEP_0204723258
Synedra acus AFS18271.1
Phaeodactylum tricornutum ACJ65494.1 SIT3
Extubocellulus spinifer CAMPEP_0178526712
Prymnesium neolepis SIT1
Scyphosphaera apsteinii SIT1
Stephanoeca diplocostata CCL97462.1 SITa
Stephanoeca diplocostata CCL97464.1 SITc
Stephanoeca diplocostata CCL97463.1 SITb
centric diatoms
pennate diatoms
chrysophytes
pennate diatoms
haptophytes
choanoflagellates
0.2
B
SIT N-terminal
Thalassiosira pseudonana ABB81825.1
Thalassiosira pseudonana ABB81826.1
Cylindrotheca fusiformis AAC49653.1
Phaeodactylum tricornutum XP 002184375.1
Scyphosphaera apsteinii SIT1
Prymnesium neolepis SIT1
Stephanoeca diplocostata CCL97464.1
SIT C-terminal
Thalassiosira pseudonana ABB81825.1 C
Thalassiosira pseudonana ABB81826.1 C
Cylindrotheca fusiformis AAC49653.1 C
Phaeodactylum tricornutum XP 002184375.1 C
Prymnesium neolepis SIT1 C
Scyphosphaera SIT1 C
Stephanoeca diplocostata CCL97464.1 C
SITL clade II
Durinskia baltica CAMPEP 0170251130
Florenciella parvula CAMPEP 0119469700
Ceratium fusus CAMPEP 0172820126
SITL clade I
Coccolithus braarudii CAMPEP 0183352566
Scyphosphaera apsteinii SITL1
Ammonia sp.CAMPEP 0197048128
Rosalina sp.CAMPEP 0201589896
Ammonia sp.CAMPEP 0197072360
Molgula tectiformis CJ335150.1
Calanus finmarchicus GBFB01130299.1
Capitella teleta ELT99670.1
Platynereis dumerilii JZ419731.1
0.2

Supplementary Figure 5: Phylogenetic analyses of Si uptake mechanisms in coccolithophores

A) A maximum likelihood phylogenetic tree demonstrating the relationship between haptophyte SITs and those from stramenopiles and choanoflagellates. The SITs from stramenopiles, choanoflagellates and prymnesiophytes form distinct monophyletic clades. Final alignment size was 179 amino acids, as only partial sequences are available for the chrysophytes. A tree generated from full-length SIT sequences (final alignment size 412 amino acids) gave identical topologies with regard to the major groups (not shown). Bootstrap values >70% (100 bootstraps) and Bayesian posterior probabilities >0.85 (10^7 generations) are shown above nodes. **B)** A maximum likelihood phylogenetic tree based on an alignment of selected SITL proteins with the N- and C- terminal domains of SITs. The two domains of the SITs form strongly supported monophyletic clades supporting a single origin for SIT gene duplication.

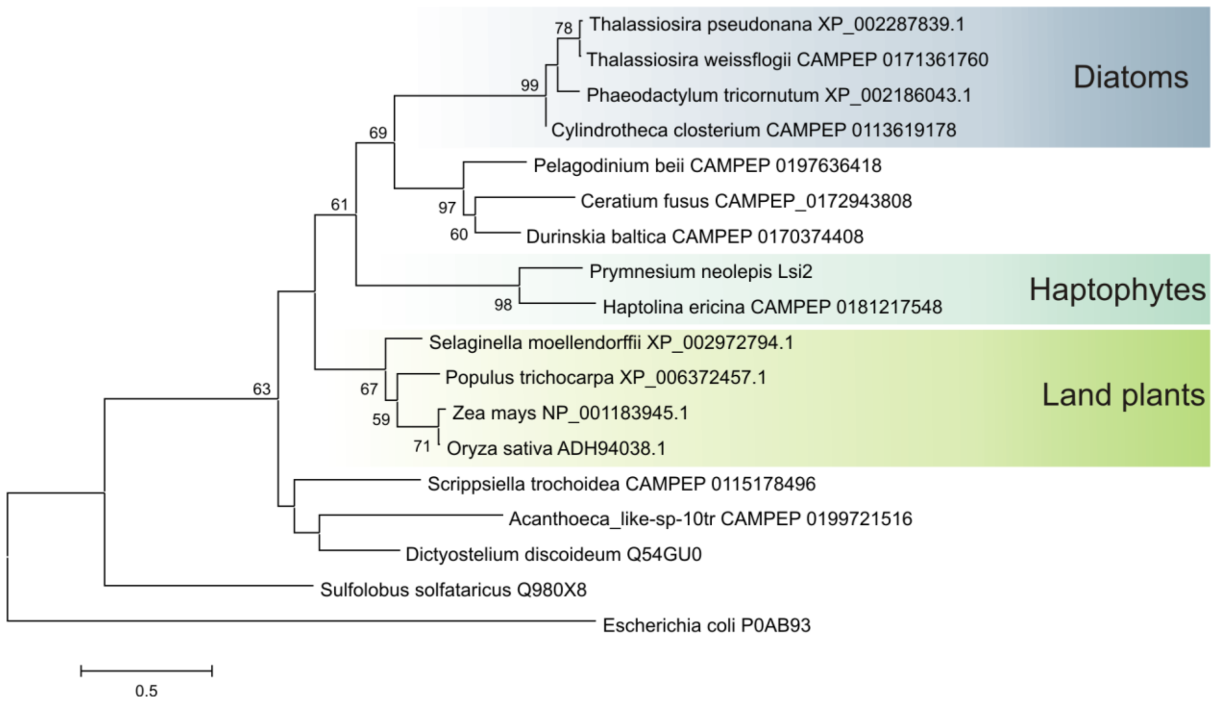

Supplementary Figure 6: Phylogenetic tree of Lsi2 Si efflux protein

A maximum likelihood phylogenetic tree is shown for Lsi2, the transporter responsible for Si efflux from some land plant cells. Lsi2 is present in the siliceous haptophyte *P. neolepis*, but was not found in any coccolithophores. Haptophyte Lsi2 sequences form a strongly supported monophyletic clade. Final alignment size was 188 amino acids. Bootstrap values >50% (100 bootstraps) are shown above nodes.

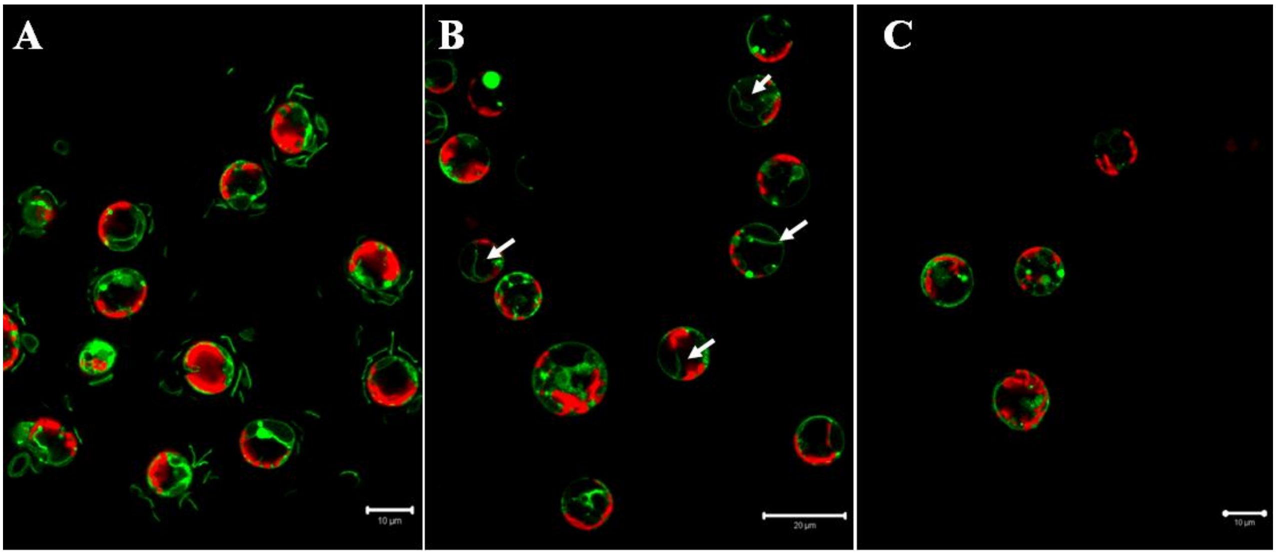

Supplementary Figure 7: Ge inhibits silica scale formation in *P. neolepis*

A) Confocal microscopy of *P. neolepis* cells loaded with the fluorescent dye HCK-123 for 24 h. The dye is incorporated into newly formed silica scales, which can be observed around each cell. Bar = 10 µm. B) Silica scale secretion is completely inhibited in *P. neolepis* cells treated with 10 µM Ge for 24 h. Some internal scales can be observed (arrowed). Bar = 20 µm C) Cells treated with 50 µM Ge for 24 h. Bar = 10 µm.

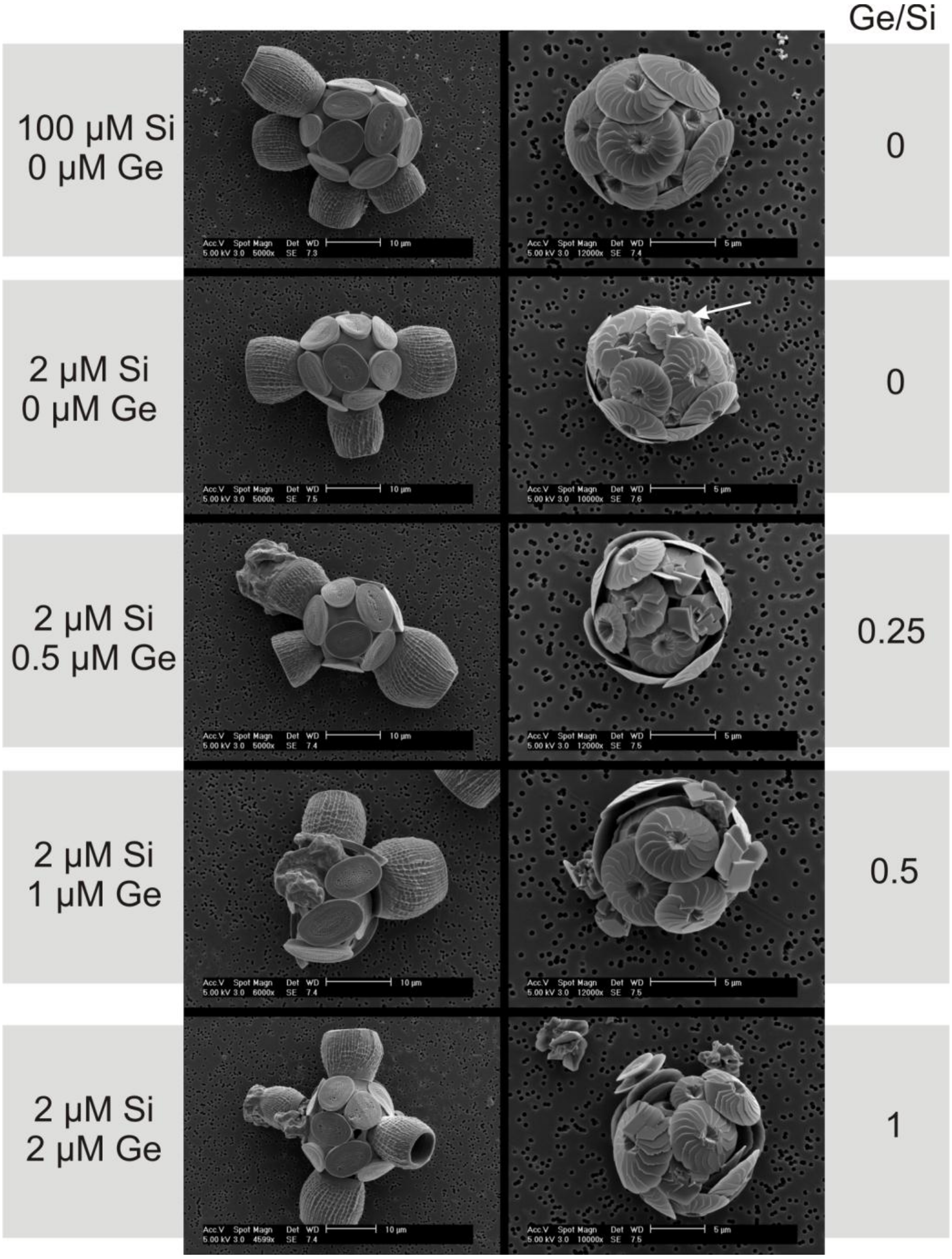

Supplementary Figure 8: The disruption of calcification by Ge is dependent on the Ge/Si ratio in *S. apsteinii* and *C. leptoporus*

A) SEM images of *S. apsteinii* and *C. leptoporus* cells treated with 0, 0.5, 1 or 2 µM Ge for 48 h in seawater containing 2 µM Si. Highly aberrant coccolith morphology can be observed at the higher Ge/Si ratios, whilst more subtle defects in coccolith morphology are present at 0.25 Ge/Si. Note also that *C. leptoporus* cells grown in the absence of Ge at 2 µM Si exhibit

'blocky' liths (arrowed) similar to those observed in *C. braarudii* cells. Defects in coccolith morphology are not observed in cells grown in Si replete media (100 µM).

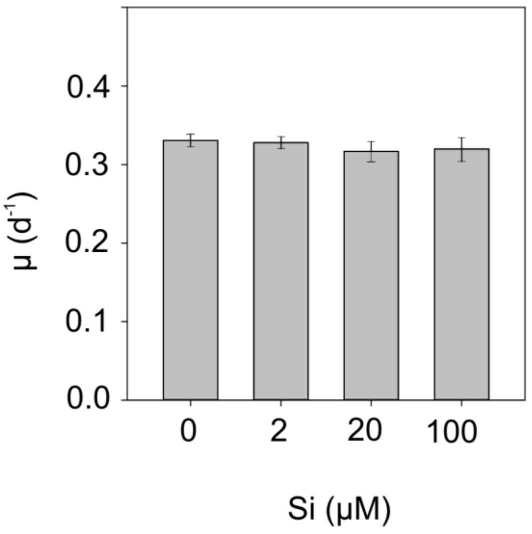

Supplementary Figure 9: Growth of Si-limited *C. braarudii* cultures

Mean specific growth rate of *C. braarudii* cells grown at 0, 2, 20 and 100 μM Si for 8d. No significant differences were identified between treatments (one-way ANOVA, n= 3), suggesting that the effects of Si-limitation on calcification in *C. braarudii* are not caused by a general disruption of cell physiology.

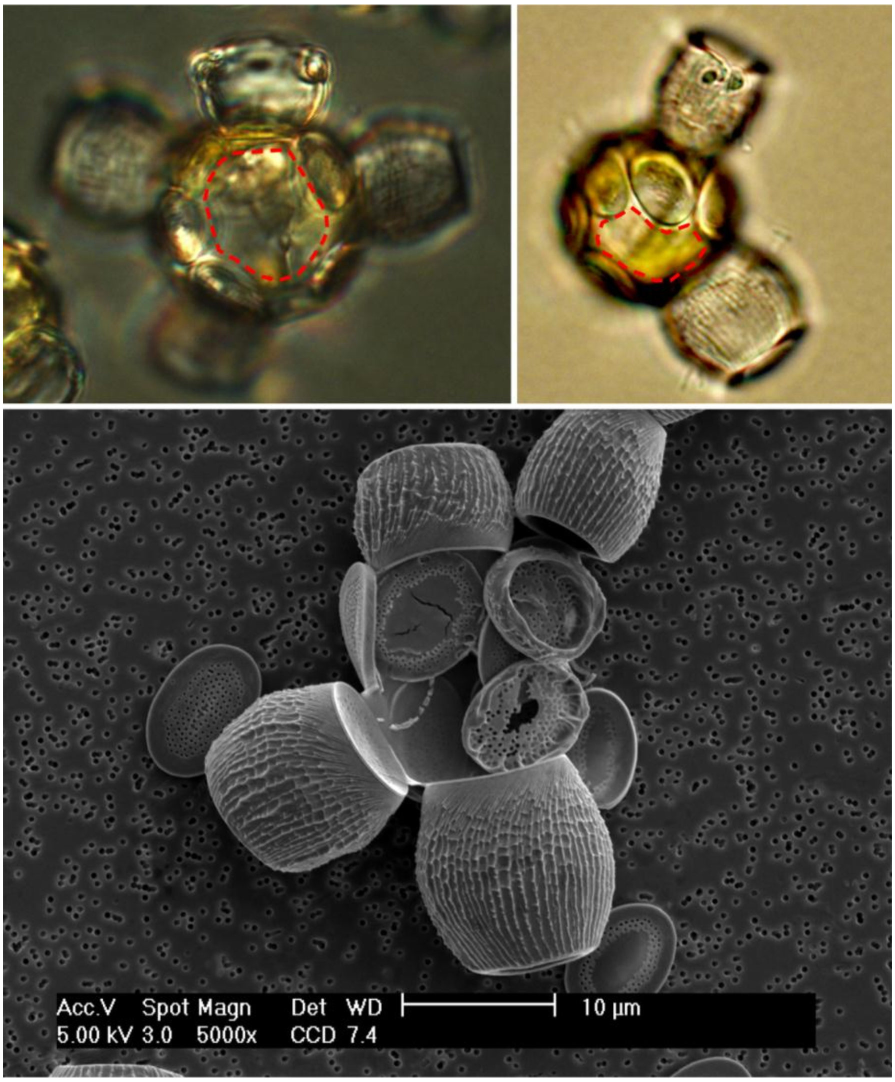

Supplementary Figure 10: Disrupted coccolith formation in *S. apsteinii* cells grown in low Si

A) Bright-field microscopy (upper) and SEM images (lower) of *S. apsteinii* cells grown in low Si media (<0.1 µM) for 8 d. Many cells exhibit missing muroliths (areas highlighted by red dotted lines), which is highly unusual compared to cells grown in Si-replete media. The absence of muroliths causes the coccospheres to collapse during sample preparation for SEM imaging. The SEM image demonstrates that the formation of both muroliths and lopadoliths are severely disrupted, with many examples of coccoliths failing to assemble completely.

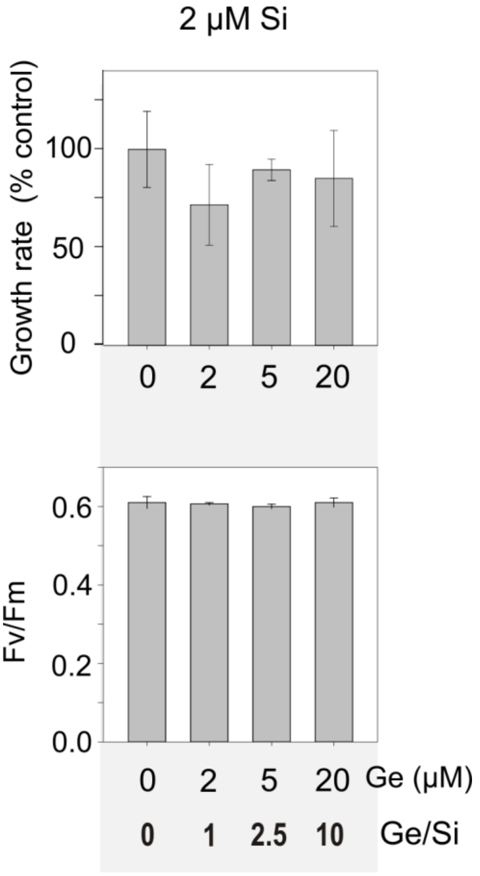

Supplementary Figure 11: *P. carterae* is insensitive to Ge

P. carterae cells were treated with 0, 2, 5 or 20 µM Ge for 48 h in seawater containing 2 µM Si. Growth and photosynthetic efficiency were not affected by Ge/Si ratios up to 10. Visual inspection by light microscopy did not indicate any defects in calcification. n=3. Error bars denote standard errors.

Protein	Organism	Description	***P. neolepis***	***E. huxleyi***	***T. pseudonana***
**Silica-associated proteins**					
frustulin	diatoms	Acidic and cys- rich			●
pleuralin	diatoms	Pro/Ser/Cys/Asp-rich			
silaffin	diatoms	Lys/Ser-rich phospho-proteins			●
cingulin	diatoms	Lys/Ser-rich proteins			●
silacidin	diatoms	Ser/Asp/Glu-rich proteins			●
silicatein[1]	sponges	related to cathepsin proteases			
silintaphin	sponges				
LPCL1	*P. neolepis*		●		
**Silicon transporters**					
SIT	diatoms	Na^+-coupled Si transporter	●		●
Lsi1[2]	land plants	aquaporin-like protein			
Lsi2[3]	land plants	Si efflux protein	●	?	●
**Polyamine synthesis**					
Adomet decarboxylase			●	●	●
Ornithine decarboxylase			●	●	●
Spermidine synthase			●	●	●
Thermospermine synthase			●	●	●
Carbomyl phosphate synthase			●	●	●

[1] Sequences exhibiting similarity to cathepsins are present in *P. neolepis* but these lack the Cys to Ser replacement at the active site found in all silicateins (*24*)
[2] A sequence exhibiting similarity to the NIP 2 class of plant aquaporins is present in *P. neolepis* but this does not contain the conserved selectivity filter of plant silicon transporters (GSGR) (*18*)
[3] A protein is present in *E. huxleyi* that exhibits some similarity to the C-terminal region of Lsi2, but this protein only has 5 transmembrane domains and is distinct from all other Lsi2 homologues, which have 10 transmembrane domains.

Supplementary Table 1: Comparison of silicification mechanisms within phytoplankton

The transcriptome of *P. neolepis* was searched for the presence of known silicification-associated proteins (black circles represent positive hits). The presence of these genes in the genomes of the coccolithophore *E. huxleyi* and the diatom *T. pseudonana* is also indicated. Note that polyamine synthesis is highly conserved amongst eukaryotes and does not indicate a role in silicification.

MMET ID	Phylum	Class	Family	Genus	Species	Strain	SIT	SITL	LPCL1	Lsi2
MMETSP1464	Haptophyta	Pavlovophyceae	Pavlovaceae	*Exanthemachrysis*	*gayraliae*	RCC1523				
MMETSP1381	Haptophyta	Pavlovophyceae	Pavlovaceae	*Pavlova*	*sp.*	CCMP459				
MMETSP1463	Haptophyta	Pavlovophyceae	Pavlovaceae	*Pavlova*	*lutheri*	RCC1537				
MMETSP1466	Haptophyta	Pavlovophyceae	Pavlovaceae	*Pavlova*	*gyrans*	CCMP608				
Pavlova-sp-CCMP459	Haptophyta	Pavlovophyceae	Pavlovaceae	*Pavlova*	*sp.*	CCMP459				
MMETSP1334	Haptophyta	Prymnesiophyceae	Calcidiscaceae	*Calcidiscus*	*leptoporus*	RCC1130		●		
MMETSP0164_2	Haptophyta	Prymnesiophyceae	Coccolithaceae	*Coccolithus*	*braarudii*	PLY182g		●		
MMETSP1333	Haptophyta	Prymnesiophyceae	Pontosphaeraceae	*Scyphosphaera*	*apsteinii*	RCC1455	●	●		
Pleurochrysis-carterae-	Haptophyta	Prymnesiophyceae	Pleurochrysidaceae	*Pleurochrysis*	*carterae*	CCMP645				
Isochrysis-galbana-	Haptophyta	Prymnesiophyceae	Isochrysidaceae	*Isochrysis*	*galbana*	CCMP1323				
Isochrysis-sp-	Haptophyta	Prymnesiophyceae	Isochrysidaceae	*Isochrysis*	*sp*	CCMP1244				
Isochrysis-sp-	Haptophyta	Prymnesiophyceae	Isochrysidaceae	*Isochrysis*	*sp.*	CCMP1324				
Emiliania-huxleyi-374	Haptophyta	Prymnesiophyceae	Noelaerhabdaceae	*Emiliania*	*huxleyi*	374				
Emiliania-huxleyi-379	Haptophyta	Prymnesiophyceae	Noelaerhabdaceae	*Emiliania*	*huxleyi*	379				
Emiliania-huxleyi-	Haptophyta	Prymnesiophyceae	Noelaerhabdaceae	*Emiliania*	*huxleyi*	CCMP370				
Emiliania-huxleyi-	Haptophyta	Prymnesiophyceae	Noelaerhabdaceae	*Emiliania*	*huxleyi*	PLY M219				
Gephyrocapsa-oceanica-	Haptophyta	Prymnesiophyceae	Noelaerhabdaceae	*Gephyrocapsa*	*oceanica*	RCC1303				
Prymnesium neolepis*	Haptophyta	Prymnesiophyceae	Prymnesiaceae	*Prymnesium*	*neolepis*	PZ241	●		●	●
Prymnesium-parvum	Haptophyta	Prymnesiophyceae	Prymnesiaceae	*Prymnesium*	*parvum*	Texoma1				
CCMP1757	Haptophyta	Prymnesiophyceae	Prymnesiaceae	*Chrysochromulina*	*polylepis*	CCMP1757				
MMETSP0286	Haptophyta	Prymnesiophyceae	Prymnesiaceae	*Chrysochromulina*	*polylepis*	UIO037				
MMETSP0287	Haptophyta	Prymnesiophyceae	Prymnesiaceae	*Chrysochromulina*	*rotalis*	UIO044	○			
MMETSP1335	Haptophyta	Prymnesiophyceae	Prymnesiaceae	*Chrysoculter*	*rhomboideus*	RCC1486				
MMETSP1094	Haptophyta	Prymnesiophyceae	Prymnesiaceae	*Haptolina*	*brevifilum*	UTEX LB 985	○			
MMETSP1096	Haptophyta	Prymnesiophyceae	Prymnesiaceae	*Haptolina*	*ericina*	CCMP281				●
MMETSP1474	Haptophyta	Prymnesiophyceae	Prymnesiaceae	*Imantonia*	*sp.*	RCC918				
MMETSP1100	Haptophyta	Prymnesiophyceae	Phaeocystaceae	*Phaeocystis*	*antarctica*	Caron Lab				
MMETSP1444	Haptophyta	Prymnesiophyceae	Phaeocystaceae	*Phaeocystis*	*antarctica*	CCMP1374				
MMETSP1465	Haptophyta	Prymnesiophyceae	Phaeocystaceae	*Phaeocystis*	*cordata*	RCC1383				
MMETSP1162	Haptophyta	Prymnesiophyceae	Phaeocystaceae	*Phaeocystis*	*Sp*	CCMP2710				
MMETSP1178	Haptophyta	Prymnesiophyceae	Unknown	*Undescribed*	*Undescribed*	CCMP2000				

Supplementary Table 2: Silicification-related proteins in haptophyte transcriptomes

Closed circles represent SIT, SITL, LPCL1 and Lsi2 proteins identified in haptophyte transcriptomes. Two further SIT sequences found in *Chrysochromulina rotalis* and *Haptolina brevifilum* (previously *Chrysochromulina brevifilum*) are shown with open circles. These sequences were not closely related to the other haptophyte SITs. The putative SIT from *Chrysochromulina rotalis* (CAMPEP_0115890138) exhibited 100% amino acid identity to a SIT from the diatom *Skeletonema costatum* (ABB81824.1), suggesting a contaminant origin for this sequence. The putative SIT from *Haptolina brevifilum* (CAMPEP_0174702132) was highly similar to SITs from the choanoflagellates, *Stephanoeca diplocostata*, *Acanthoeca* and *Diaphanoeca grandis* (BLASTP E value $2e^{-170}$, 56-59% identity at the amino acid level), also suggesting that a contaminant origin is the most likely explanation. All further analyses therefore focused on the SITs identified in the biomineralising haptophytes. Expression of these SITs was verified by RT-PCR, using independent cultures and/or independently isolated strains from those used to generate the transcriptome. CAMPEP IDs of proteins listed in table: *S. apsteinii* SIT1 CAMPEP_0119335116; *S. apsteinii* SITL1 CAMPEP_0119299848; *C. braarudii* SITL1 CAMPEP_0183352566. Accession numbers: *P. neolepis* SIT1 KP793098, LPCL1 KP793099, *C. leptoporus* SITL1 KR677451. *All transcriptomes can be accessed through the MMETSP database, except *P. neolepis*, which was sequenced and assembled by Genoscope.

Gene product	Species	Primer name	Sequence
SIT	*Prymnesium neolepis*	PnSIT1_F1<br>PnSIT1_R1	CAGCGTGATTCTCCACATTTC<br>GAAATGTGGAGAATCACGCTG
SIT	*Scyphosphaera apsteinii*	SaSIT1_F2<br>SaSIT1_R2	TAAGCCTCTCTCAGCAGCTG<br>GCGGCGCGTATAAAGGTATA
SITL	*Coccolithus braarudii*	CpSITL1_F2<br>CpSITL1_R2	CATGGGCACAGCTTCTTCT<br>GCTTGAACTGGAGGAGCAG
SITL	*Calcidiscus leptoporus*	ClSITL1_F1<br>ClSITL1_R2	TCTTCTCCTCCCTCCTCCTC<br>TTGAGGACTCGCGGGAAC
LPCL	*Prymnesium neolepis*	PnLPCL1_F1<br>PnLPCL1_R1	GTTGCAAGTACTTCATGCCAAGC<br>GCATGAAGTAGTCGCAGCCCTCA

Supplementary Table 3: Primers used for RT-PCR of Si-related genes in haptophytes
